# Examining Farnesyltransferase Interaction With Cell‐Permeable CaaX Peptides and the Role of the CaaX Motif in Biological Activity

**DOI:** 10.1002/psc.70009

**Published:** 2025-03-10

**Authors:** Merlin Klußmann, Jan Reuter, Christian Werner, Ines Neundorf

**Affiliations:** ^1^ Institute for Biochemistry, Department of Chemistry and Biochemistry University of Cologne Cologne Germany

**Keywords:** CaaX motif, cell‐permeable CaaX peptides, competition, farnesyltransferase, inhibition, prenylation

## Abstract

Recently, we presented cell‐permeable CaaX peptides as versatile tools to study intracellular prenylation of proteins. These peptides consist of a cell‐penetrating peptide (CPP) and a *C*‐terminal CaaX motif derived from Ras proteins and demonstrated high cellular accumulation and the ability to influence Ras signaling in cancerous cells. Here, we aimed to gain a deeper insight into how such cell‐permeable CaaX peptides, particularly the KRas4B‐derived CaaX‐1 peptide, interact with farnesyltransferase (FTase) and likely influence further intracellular processes. We show that CaaX‐1 is farnesylated by FTase ex cellulo and that an intact CaaX motif is required for modification. A competition experiment revealed a slower farnesylation of CaaX‐1 by FTase compared to a CaaX motif‐containing control peptide. CaaX‐1 inhibited farnesylation of this control peptide at considerably lower concentrations; thus, a higher affinity for FTase is hypothesized. Notably, AlphaFold3 not only predicted interactions between CaaX‐1 and FTase but also suggested interactions between the peptide and geranylgeranyltransferase type I. This finding encourages further investigation, as cross‐prenylation is a well‐known drawback of FTase inhibitors. Our results are further evidence for the usefulness of CaaX peptides as tools to study and manipulate the prenylation of proteins. They offer real potential for the development of novel inhibitors targeting the prenylation pathway.

## Graphical abstract

This work examines the interaction between farnesyltransferase (FTase) and cell‐permeable CaaX peptides. Particularly, peptide CaaX‐1 is presented as efficient binder to FTase capable to inhibit further interaction of other CaaX motif–bearing peptides. Moreover, biological effects require an intact CaaX motif. Thus, we suggest CaaX peptides as versatile tools to study protein prenylation.

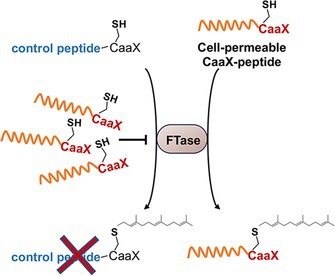

**Abbreviations**
ACN: acetonitrile
CF: 5(6)‐carboxyfluorescein
CPP: cell‐penetrating peptide
FA: formic acid
FPP: farnesyl diphosphate
FTI: farnesyltransferase inhibitor
GGTase: geranylgeranyltransferase
*h*/*r*FTase: human/rat farnesyltransferase
ipTM: interface predicted template modeling
MCS: multiple cloning site
pTM: predicted template modeling
TFA: trifluoroacetic acid

## 1 Introduction

Protein prenylation is a posttranslational lipidation in eukaryotes that enables localization to certain membranes, such as the plasma membrane [1, 2, 3, 4, 5, 6]. The process is catalyzed by prenyltransferases including farnesyltransferase (FTase) and geranylgeranyltransferase Types I, II, and III (GGTase I–III) [7, 8, 9, 10, 11, 12, 13]. However, the majority of prenylations are mediated by FTase and GGTase I, both consisting of an α‐subunit and a catalytic β‐subunit [14, 15]. Prenylation occurs at a *C*‐terminal CaaX motif in which the cysteine is modified by either farnesyl or geranylgeraniol [16, 17, 18]. The type of prenylation that takes place is mainly determined by the *C*‐terminal variable amino acid X. Proteins bearing an isoleucine, leucine, or phenylalanine at this position are usually geranylgeranylated by GGTase I, while alanine, methionine, or serine leads to farnesylation by FTase [7, 8, 9, 19, 20, 21]. In fact, many proteins require prenylation for proper localization and biological activity, as for instance, Ras proteins [22, 23]. They function as signal transductors, and when they are “switched on” by incoming signals, they activate signaling cascades that regulate cell proliferation, differentiation, and survival [10, 24]. However, Ras proteins are often associated to cancer as mutations in Ras genes can lead to their permanent activation also in the absence of extracellular signals [10, 25]. For a long time, Ras‐mediated cancer was termed “undruggable” [26, 27], but recently, significant progress has been made in this field as several KRas G12C inhibitors, for example, sotorasib (AMG‐510, Amgen) [28, 29], adagrasib (MRTX‐849, Mirati) [30, 31], and JNJ‐74699157 (ARS‐3248, J&J) [32, 33, 34], were developed. These substances bind covalently to the mutated cysteine in KRas G12C forcing a conformational change of the switch regions and increasing the affinity of KRas to GDP instead of GTP. Owing to their high anticancer activity, these inhibitors were already approved by the FDA or are currently in clinical development [34, 35]. Moreover, they serve as a basis for the further development of covalent inhibitors targeting other KRas variants, such as the highly present KRas G12D mutant. However, recent progress failed owing to the difficulty in targeting the mutated amino acids [36].

Besides direct targeting of Ras proteins, there also exist indirect targeting strategies to combat Ras‐associated cancer. For example, limiting the transport of Ras proteins to the plasma membrane by PDEδ inhibition is one possibility. PDEδ is a chaperone that binds to and transports prenylated proteins [36]. Indeed, NHTD is a recently developed PDEδ inhibitor provoking loss of plasma membrane localization and antitumor activity in xenograft and KRas mutant mice [36, 37]. Another point of attack is the farnesylation process itself. Preventing farnesylation of Ras proteins would impair their plasma membrane localization and activity. In fact, FTase inhibitors (FTIs), which included various peptidomimetic compounds [38, 39, 40, 41], showed promising results in preclinical studies but had only limited effects in clinical studies. For instance, lipidation of NRas and KRas was rescued by GGTase I cross‐prenylation. In contrast, HRas is exclusively prenylated by FTase and might be therefore a better target for FTIs [36, 42, 43, 44, 45, 46, 47].

Recently, we highlighted cell‐permeable CaaX peptides comprising the cell‐penetrating peptide sC18* and *C*‐terminal sequences of Ras proteins including the CaaX motif [48]. These peptides showed high cellular uptake and cytotoxicity to cancer cells, which was dependent on the CaaX motif. In fact, we proved that a serine‐substituted SaaX motif significantly decreased their cellular effects. Studies with the KRas4B‐derived CaaX‐1 peptide (GLRKRLRKFRNK‐SKTKCVIM) revealed that this peptide altered downstream signaling of Ras proteins in KRas mutant pancreatic cancer cells. Because a pull‐down experiment revealed that CaaX‐1 interacts specifically with endogenous prenyltransferases, we herein aimed to gain more insights into these interactions between CaaX peptides and FTase. Our findings let assume that CaaX‐1 is farnesylated by FTase in vitro and that the peptide can prevent farnesylation of a control peptide (GCVLS). Moreover, we suggest that both the farnesylation and the efficient cellular uptake of CaaX‐1 peptides into HeLa cells require an intact CaaX motif.

## 2 Materials and Methods

### 2.1 Peptide Synthesis and Purification

All peptides were synthesized by SPPS following the Fmoc/tBu strategy using an automated Syro I peptide synthesizer (MultiSynTech). As solid support, preloaded Wang resins or Rink amide resins were used. CaaX‐1 was synthesized as previously described [48]. For intracellular visualization, peptides were *N*‐terminally coupled on the resin with 5,6‐carboxyfluorescein (CF) using 5 equivalents (eq.) each of CF, Oxyma, and *N*,*N*′‐diisopropylcarbodiimide (DIC) at room temperature overnight. For the synthesis of the dansylated CaaX‐1 peptide, Fmoc‐L‐Lys (Dde)‐OH was introduced to selectively couple the dansyl group to the desired lysine side chain. For this, the resin was shaken with 2 eq. of dansyl chloride and 1 eq. of DIPEA in *N*‐methyl‐2‐pyrrolidone (NMP) at room temperature for 2 h. Peptides were cleaved from the resin by incubation with a TFA/thioanisol/1,2‐ethanedithiol (90:7:3, v/v/v) mixture at room temperature for 3 h under shaking conditions. Subsequently, peptides were precipitated in ice‐cold diethyl ether and lyophilized. Peptides were purified by preparative RP‐HPLC (Elite LaChrom, Hitachi, Chiyoda, Japan) on a VP 250/16 Nucleodur 100‐5 C18ec column (Macherey‐Nagel, Düren, Germany) using a linear gradient from 10% to 45% or 40% to 80% B in A (A: 0.1% TFA in H_2_O, B: 0.1% TFA in ACN) in 45 min. Qualitative analysis was performed by RP‐HPLC (Hewlett Packard Series 1100, Agilent; column: EC 125/4.6 NUCLEODUR 100‐5 C18ec, Macherey‐Nagel, Düren, Germany) using a linear gradient of 10%–60% or 20%–70% B in A in 15 min (A: H_2_O with 0.1% formic acid (FA), B: ACN with 0.1% FA) and subsequent electrospray‐ionization mass spectrometry analysis (LTQ‐XL, Thermo Scientific, Waltham, Massachusetts, USA). UV spectra were generated by analytical RP‐HPLC using a linear gradient of 10%–60% or 20%–70% B in A in 15 min (A: H_2_O with 0.1% TFA, B: ACN with 0.1% TFA). See Table S1 for a summary of all synthesized peptides.

### 2.2 Recombinant Expression of Human FTase (*h*FTase)

To recombinantly express *h*FTase, the genes for the two subunits of FTase (*FNTA* and *FNTB*) were subcloned into the same vector (pCDFDuet1, Novagen) containing two multiple cloning sites (MCSs). MCS I also encodes a His_6_‐tag enabling purification via Ni‐NTA affinity chromatography. First, the *FNTA* gene was subcloned into MCS I. For this, the *FNTA* gene (NM_002027, Origene) was amplified by PCR using 5′‐TTAAGAATTCATGGCGGCCACCGAG‐3′ (forward) and 5′‐GCTATAAGCTTTTATTGCTGTACATTTGTTGGTGAGTC‐3′ (reverse) as primers. The primer contained restriction sites of the EcoRI and HindIII, which also have restriction sites within the MCS I of the vector. After digestion of the *FNTA* gene and the pCDFDuet‐1 vector, the gene was ligated into the vector. To bring the *FNTA* gene in reading frame with the His_6_‐tag, a mutagenesis was performed using the QuickChange II site‐directed mutagenesis kit (Aligent) and 5′‐ATCACCATCATCACCACAAGCCAGGATCCGAATTC‐3′ (forward) and 5′‐GAATTCGGATCCTGGCTTGTGGTGATGATGGTGAT‐3′ (reverse) as primers. Amplification of the vector was performed in *Escherichia coli* DH5α using 50‐μg/mL streptomycin for selection. Next, the *FNTB* gene was subcloned into the MCS II of the pCDFDuet‐1‐*FNTA* vector. For this, *FNTB* was amplified using the primers 5′‐GTATCCTCGAGATGGCTTCTCCGAGT‐3′ (forward) and 5′‐CGCGTTAATTAACTAGTCGGTTGCAGGCT‐3′ (reverse), which contained the restriction sites for XhoI and PacI. After digestion, the *FNTB* gene was ligated into the MCS II of the vector. Because there was an additional start codon within MCS II between the promoter and the start codon of the gene, a further mutagenesis was performed using 5′‐AAGAAGGAGATATACATAGGGCAGATCTCAATTGGATATCG‐3′ (forward) and 5′‐CGATATCCAATTGAGATCTGCCCTATGTATATCTCCTTCTT‐3′ (reverse) as primers.

Expression of the two subunits of FTase was performed in competent *E. coli* Rosetta (DE3)pLysS in LB medium using chloramphenicol and streptomycin (50 μg/mL) as antibiotics. Gene expression was induced by 0.5‐mM IPTG and 0.5‐mM ZnSO_4_ and carried out at 18°C and 210 rpm overnight. Cells were harvested by centrifugation at 4°C and 5000 rpm for 20 min. Cell lysis was carried out in lysis buffer (50‐mM Tris pH 7.7, 200‐mM NaCl, 5.0‐μM ZnSO_4_, 5.0‐mM MgCl_2_, 20‐mM imidazole, 1.0‐mM DTT, 20‐μg/mL DNase I, a spatula tip of lysozyme [Sigma Aldrich], and cOmplete EDTA‐free protease inhibitor cocktail) at 4°C for 45 min and subsequent sonification (2 s on, 4 s off, 2 min, 35% amplitude) at 4°C. Cell debris were removed by centrifugation at 30,000 rpm and 4°C for 45 min.

For protein purification, Ni‐NTA affinity chromatography was performed using a linear gradient of 0%–100% elution buffer (50‐mM Tris HCl pH 7.7, 200‐mM NaCl, 5‐μM ZnCl_2_, 5‐mM MgCl_2_, 250‐mM imidazole, 1‐mM DTT) in purification buffer (50‐mM Tris HCl pH 7.7, 200‐mM NaCl, 5‐μM ZnCl_2_, 5‐mM MgCl_2_, 20‐mM imidazole, 1‐mM DTT) using a 5‐mL column with a flow rate of 0.8 mL/min.

The buffer of the pooled fractions of the peaks were exchanged by storage buffer (50‐mM Tris HCl pH 7.7, 200‐mM NaCl, 5‐μM ZnCl_2_, 5‐mM MgCl_2_, 1‐mM DTT) and concentrated by Amicon Ultra Centrifugal Filter with 30‐kDa pore size.

### 2.3 Coomassie‐Stained SDS‐PAGE and Western Blot

All samples for SDS‐PAGE were mixed with Laemmli buffer and incubated at 95°C for 5 min. Then, samples were separated by SDS‐PAGE. For Coomassie staining, gels were incubated in a Coomassie staining solution (1.5‐g Coomassie brilliant blue R‐250, 225‐mL H_2_O, 225‐mL MeOH, 50‐mL glacial acetic acid) at room temperature for 1 h, and the background was subsequently destained by destaining solution (10% EtOH, 5% glacial acetic acid in H_2_O).

For Western blot analysis, proteins from the SDS–polyacrylamide gels were transferred onto an PVDF membrane. The membrane was first activated with MeOH, and the proteins were subsequently transferred onto the membrane using a semi‐dry blotting method. Then, the membrane was blocked in 5% milk powder in PBS‐T (0.1% Tween‐20 in PBS) at room temperature for 1 h and incubated with the primary antibody in 5% milk powder in PBS‐T at 4°C overnight. The membrane was washed with PBS‐T and incubated with the secondary antibody in 5% milk powder in PBS‐T at room temperature for 1.5 h. After further washing with PBS‐T, the membrane was developed.

Primary antibodies: anti‐FNTA monoclonal (ab Cat#109738, 1:1000), anti‐FNTA (Proteintech Cat#12274‐1‐AP, 1:1000), and anti‐FNTB (ab Cat#109748, 1:1000). The manufacturers of the primary antibodies have confirmed their reactivity for *h*FTase and rat FTase (*r*FTase). Secondary antibody: anti‐rabbit‐HRP conjugate (CST Cat#7074S, 1:1000).

### 2.4 Dansyl‐Based Peptide Farnesylation Assay

Fifty micromoles of dansylated peptide, 10‐μM farnesyl diphosphate (FPP), and 50‐nM *r*FTase (Jena Bioscience, Cat#PR‐102) or *h*FTase were dissolved in 50‐mM Tris HCl pH 7.5, 5‐mM DTT, 5‐mM MgCl_2_, 10‐μM ZnCl_2_, and 0.2% *n*‐octyl‐beta‐d‐glucopyranoside and were transferred into a black bottom 96‐well plate. The change in fluorescence at 505 nm was measured after excitation at 340 nm for 200 min. Potential farnesylation of the peptide would lead to hydrophobic interactions between the dansyl and farnesyl group, which in turn would lead to a shift in fluorescence maximum at 505 nm [49].

### 2.5 Mass Spectrometry–Based Farnesylation Assay

Fifty‐micromole CaaX‐1 or scrambled control was incubated with 50‐nM recombinantly expressed *h*FTase or *r*FTase (Jena Bioscience, Cat#PR‐102) and 10‐μM farnesyl pyrophosphate (FPP) in reaction buffer (50‐mM Tris HCl pH 7.5, 5‐mM DTT, 5‐mM MgCl_2_, 10‐μM ZnCl_2_, and 0.2% *n*‐octyl‐beta‐d‐glucopyranoside) at 37°C for 18 h. Afterwards, the reaction mixture was desalted via C18 ZipTips (Merck Millipore). For this, ZipTips were washed with ACN and equilibrated in 0.1% FA in H_2_O. Then, the column was loaded with the reaction solution and subsequently washed with 0.1% FA in H_2_O and 5% MeOH in H_2_O each supplemented with 0.1% FA. Elution was performed using 50% ACN with 0.1% FA, and the eluted solution was analyzed by RP‐HPLC using a linear gradient of 10%–60% ACN in H_2_O with 0.1% FA or TFA using a flow rate of 0.6 or 1.0 mL/min, respectively, and by ESI‐MS analysis (LTQ‐XL, Thermo Scientific, Waltham, Massachusetts, USA).

### 2.6 Time‐Dependent Farnesylation Assay

Twenty‐five micromolar dan‐CaaX‐1 or dan‐GCVLS were incubated with 50‐nM *r*FTase (Jena Bioscience, Cat#PR‐102) and 20‐μM FPP in reaction buffer (50‐mM Tris HCl pH 7.5, 5‐mM DTT, 5‐mM MgCl_2_, 10‐μM ZnCl_2_, and 0.2% *n*‐octyl‐beta‐d‐glucopyranoside) at 37°C. After several time points, samples were taken and the reaction was stopped by adding FA. Samples were measured by LC‐ESI‐MS (Nexera XR 40 series HPLC [Shimadzu], MN Nucleodur 300‐5 C18ec 5 μm, 100 × 2 mm [Macherey‐Nagel], LCMS‐8060 [Shimadzu]) with a flow rate of 0.5 mL/min and Mobile Phases A and B using the profile 0–7 min, 20%–70% B; 7–7.1 min, 70%–100% B; 7.1–10 min, 100% B; 10–10.1 min, 100%–20% B; 10.1–15 min, 20% B (A: 0.1% FA in H_2_O, B: 0.1% FA in ACN). Dansylated peptides were detected by a fluorescence detector at 505 nm after excitation at 340 nm.

### 2.7 Dansyl‐Based Farnesylation Competition Assay

Twenty‐five micromolar dan‐GCVLS, different concentrations of CaaX‐1, 20‐μM FPP, and 50‐nM *r*FTase (Jena Bioscience, Cat#PR‐102) were diluted in 50‐mM Tris HCl pH 7.5, 5‐mM DTT, 5‐mM MgCl_2_, 10‐μM ZnCl_2_, and 0.2% *n*‐octyl‐beta‐d‐glucopyranoside and transferred into a black bottom 96‐well plate. The change in fluorescence at 505 nm was measured after excitation at 340 nm for 1400 s.

### 2.8 Mass Spectrometry Analysis of Farnesylation Competition

Twenty‐five micromolar dan‐GCVLS were coincubated with different concentrations of CaaX‐1, 50‐nM *r*FTase (Jena Bioscience, Cat#PR‐102), and 20‐μM FPP in reaction buffer (50‐mM Tris HCl pH 7.5, 5‐mM DTT, 5‐mM MgCl_2_, 10‐μM ZnCl_2_, and 0.2% *n*‐octyl‐beta‐d‐glucopyranoside) at 37°C. After several time points, samples were taken, and the reaction was stopped by adding FA. Samples were measured by LC‐ESI‐MS (Nexera XR 40 series HPLC [Shimadzu], MN Nucleodur 300‐5 C18ec 5 μm, 100 × 2 mm [Macherey‐Nagel], LCMS‐8060 [Shimadzu]) with a flow rate of 0.5 mL/min and Mobile Phases A and B using the profile 0–7 min, 20%–70% B; 7–7.1 min, 70%–100% B; 7.1–10 min, 100% B; 10–10.1 min, 100%–20% B; 10.1–15 min, 20% B (A: 0.1% FA in H_2_O, B: 0.1% FA in ACN). Dansylated peptides were detected by a fluorescence detector at 505 nm after excitation at 340 nm.

### 2.9 Mass Spectrometric Analysis of FTase Replacement Assay

Either 25‐μM dan‐GCVLS or 10‐μM CaaX‐1 were preincubated with 50‐nM *r*FTase (Jena Bioscience, Cat#PR‐102) and 20‐μM FPP in reaction buffer (50‐mM Tris HCl pH 7.5, 5‐mM DTT, 5‐mM MgCl_2_, 10‐μM ZnCl_2_, and 0.2% *n*‐octyl‐beta‐d‐glucopyranoside) at 37°C. After 30 min, 10‐μM CaaX‐1 or 25‐μM dan‐GCVLS, respectively, was added. After several time points, samples were taken, and the reaction was stopped by adding FA. Samples were measured by LC‐ESI‐MS (Nexera XR 40 series HPLC [Shimadzu], MN Nucleodur 300‐5 C18ec 5 μm, 100 × 2 mm [Macherey‐Nagel], LCMS‐8060 [Shimadzu]) with a flow rate of 0.5 mL/min and Mobile Phases A and B using the profile 0–7 min, 20%–70% B; 7–7.1 min, 70%–100% B; 7.1–10 min, 100% B; 10–10.1 min, 100%–20% B; 10.1–15 min, 20% B (A: 0.1% FA in H_2_O, B: 0.1% FA in ACN). Dansylated peptides were detected by a fluorescence detector at 505 nm after excitation at 340 nm.

### 2.10 Cell Culture

HeLa cells were cultured in petri dishes at 37°C, 5% CO_2_, and humidified atmosphere in RPMI 1640 supplemented with 10% FBS and 4‐mM l‐glutamine (complete medium). When cells were grown to 70%–80% subconfluency, they were split by using 0.5‐mg/mL trypsin–EDTA to detach the cells.

### 2.11 Flow Cytometry Measurement

Cellular uptake of peptides was quantified by flow cytometry. For this, 90,000 HeLa cells were seeded in a 24‐well plate and grown in complete medium at 37°C overnight. Cells were treated with 10 μM of CF‐labeled peptides in serum‐free medium (with 4‐mM l‐glutamine) for 30 min at 37°C. Afterwards, cells were washed with PBS (Sigma Aldrich), detached using phenol red‐free trypsin/EDTA (0.5 mg/mL), and resuspended in phenol red‐free Dulbecco's Modified Eagle's Medium with high glucose levels (Sigma Aldrich), 10% FBS, and 4‐mM l‐glutamine. Internalization was quantified using a Guava easyCyte flow cytometer (Merck) by analyzing 10,000 cells with a GRN‐B (523/30) laser.

### 2.12 Cytotoxicity Assay

Cytotoxicity of the peptides to HeLa cells were investigated using a resazurin‐based cytotoxicity assay (Sigma Aldrich). For this, 14,000 HeLa cells were seeded in a 96‐well plate and grown in complete medium at 37°C to 80%–90% subconfluency. Cells were treated with different peptide dilutions in serum‐free medium at 37°C for 24 h. Untreated cells served as negative control and cells treated with 70% ethanol for 10 min as positive control. Cells were washed with PBS and subsequently incubated with a 10% resazurin solution in serum‐free medium for ~1 h. To determine the viability of the cells, the fluorescence was measured at 595 nm after excitation at 550 nm using a Tecan infinite M200 plate reader.

### 2.13 In Silico Prediction of the Interaction of FTase With CaaX‐1

AlphaFold3 was used for in silico prediction of the interaction of FTase with CaaX‐1 or its scrambled control peptides [50]. The predicted Model 0 was loaded into Coot for inserting the zinc ion into the active site of FTase and for generating a PDB file. The PDB file of the model was used for generating images of the interaction points of FTase with CaaX‐1 using Pymol and ChimeraX [51, 52, 53].

## 3 Results and Discussion

### 3.1 Examining the Interaction of FTase With CaaX‐1 In Silico

**FIGURE 1:**
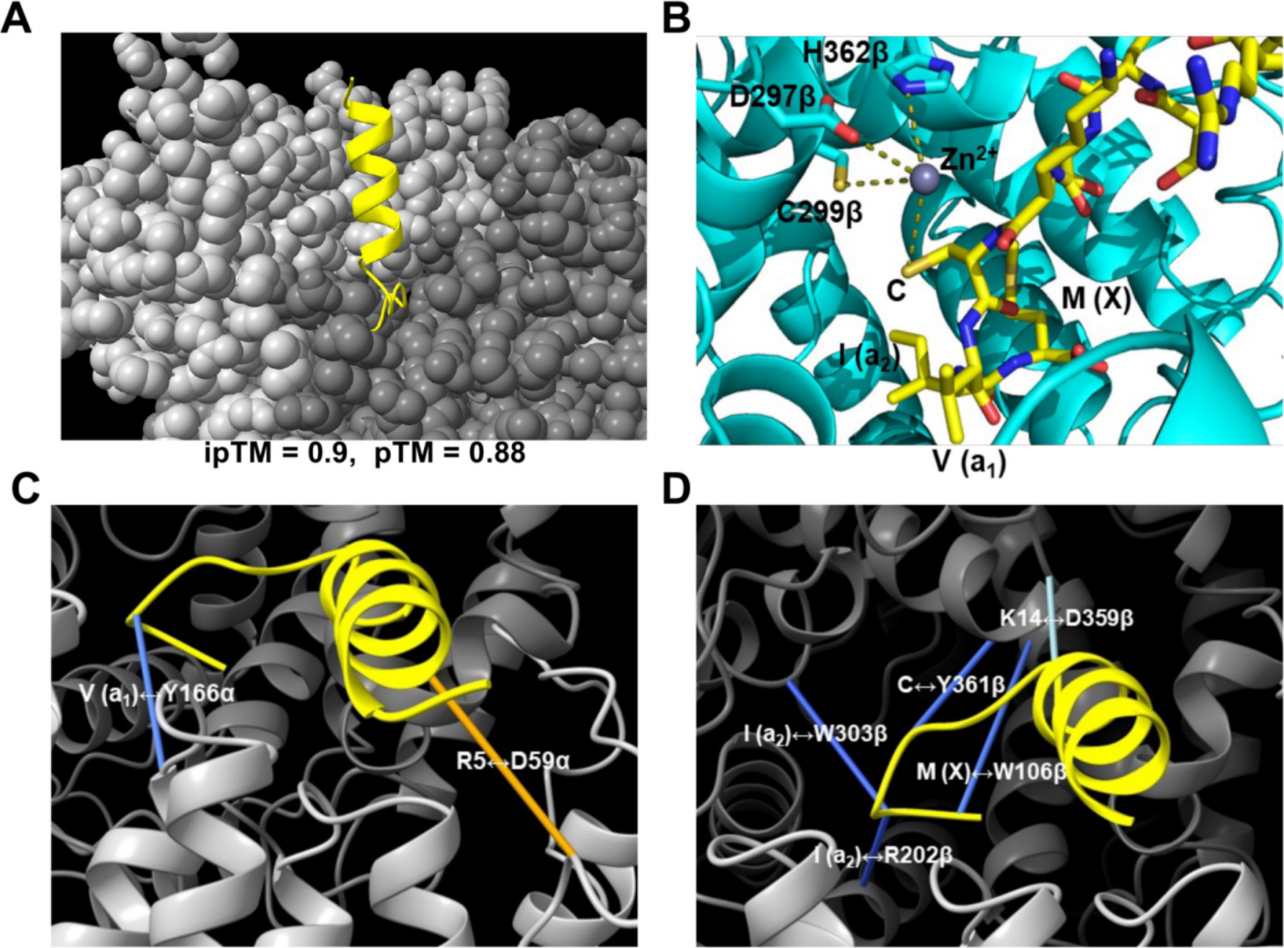
(A) AlphaFold3 predicted CaaX‐1–FTase complex. The CaaX box of CaaX‐1 occupies the active site of FTase (α‐subunit: light grey, β‐subunit: dark grey). CaaX‐1 is highlighted in yellow. Image was made with ChimeraX. (B) AlphaFold3 prediction of the interactions between CaaX‐1 (yellow) and the zinc ion (grey) complexed by the active site of FTase (cyan). AlphaFold3 prediction was processed and modified with PyMOL using PDB 1TN6. The zinc ion was inserted with Coot. AlphaFold3 predicted interactions of CaaX‐1 with the (C) α‐subunit or (D) β‐subunit of FTase. Dark blue highlights highly reliable interactions, light blue reliable interactions, and orange less reliable interactions. Images of (A), (C), and (D) were made with ChimeraX [51, 52, 53].

First, we examined if CaaX‐1 would fit into the active site of FTase. For this, we used AlphaFold3 predicting the 3D structure of the possible CaaX‐1–FTase complex [50]. A reliable structure was produced with an interface predicted template modeling (ipTM) score of 0.9 and a predicted template modeling (pTM) score of 0.88 highlighting an accurate prediction because both scores were close to 1. As can be depicted from Figure 1A, the CaaX box of CaaX‐1 occupies the active site of FTase within the interface of the α‐subunit and the β‐subunit. Notably, when inserting a zinc ion into the active site of FTase [54, 55, 56, 57, 58], interactions of the cysteine of CaaX‐1 and the zinc ion of FTase, which is complexed by D297β, C299β, and H362β, were predicted (Figure 1B). Despite, also several other interaction points were calculated when AlphaFold3 was combined with ChimeraX [51, 52, 53] including Tyr166α, Trp106β, Arg202β, and Tyr361β (Figure 1C,D). This observation actually makes sense because these residues have recently been described to be important for the activity of FTase [59, 60, 61]. Interestingly, AlphaFold3 also represented interactions between CaaX‐1 and GGTase I (Figure S10), a cross‐prenylation mechanism that was already detected in our previous studies [48]. However, we observed meaningful interactions of CaaX‐1 and FTase which we then examined more closely in the next experiments.

### 3.2 Recombinant Expression of Human FTase

**FIGURE 2:**
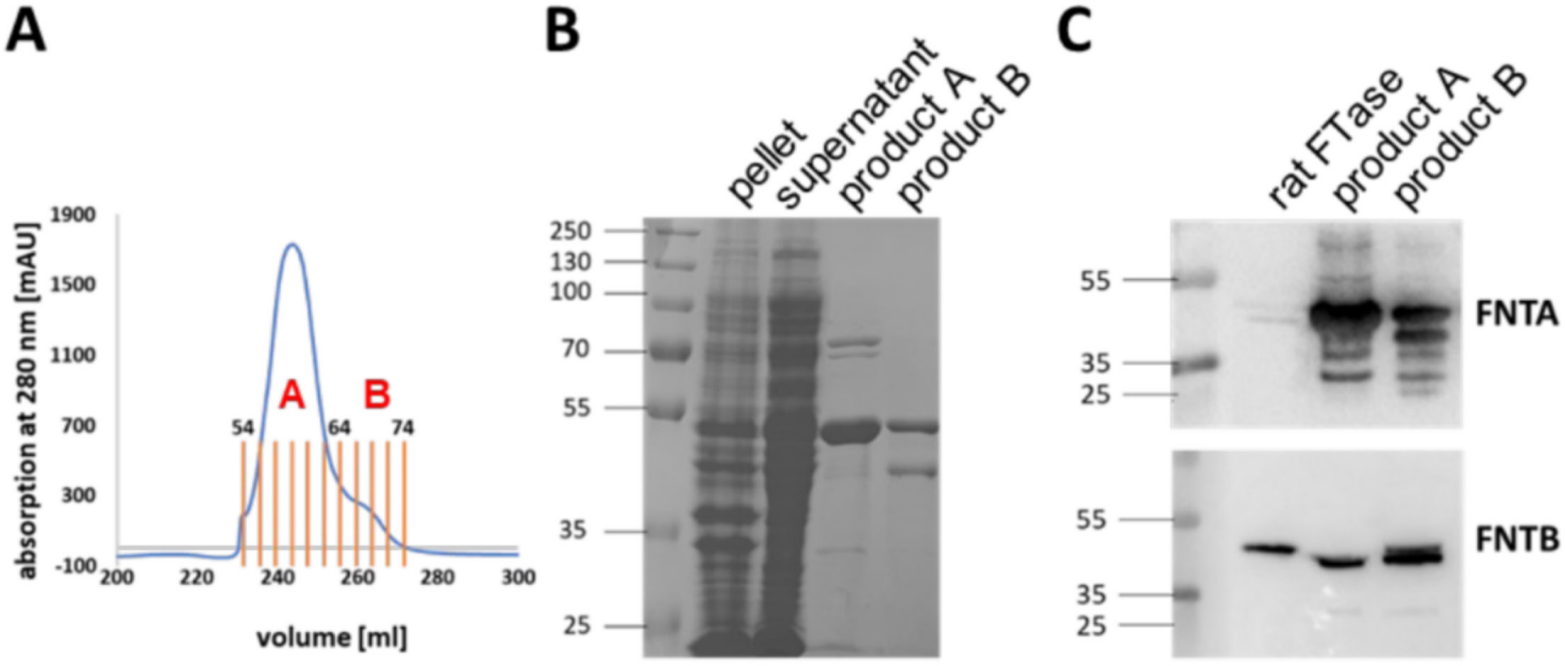
Recombinant expression and purification of *h*FTase. (A) Chromatogram of the purification of recombinantly expressed *h*FTase via Ni‐NTA affinity chromatography. (B) Coomassie‐stained SDS–polyacrylamide gel of the different purification steps. (C) Western Blots of the final samples using antibodies for the α‐subunit and β‐subunit of FTase.

For interaction studies between CaaX‐1 and FTase, we first generated *h*FTase by recombinant expression. Thus, we generated a vector to coexpress both subunits of *h*FTase [62], namely, the regulatory alpha (FNTA) and the catalytically active beta (FNTB) subunit, in *E. coli* Rosetta (DE3)pLysS, as it has been shown that stable expression of the enzyme requires simultaneous expression of both subunits [63]. Purification was realized using Ni‐NTA affinity chromatography. Ni‐NTA chromatography resulted in one broad peak (Product A) and a smaller peak (Product B) (Figure 2A).

A Coomassie‐stained SDS–polyacrylamide gel showed a relatively pure sample of Product A most likely containing the two subunits of *h*FTase (Figure 2B) [14, 15]. Western blot analysis indicated both subunits in the two fractions suggesting a successful expression (Figure 2C). However, also other bands were visible probably referring to degradation products of the α‐subunit. This was likely the result of the absence of EDTA in the lysis buffer, which was omitted because FTase bears a zinc ion within the active site [54, 55, 56, 57, 58]. Nonetheless, we used Product A for the following studies as it exhibited a higher purity and a higher concentration (22.35 mg/mL).

### 3.3 CaaX‐1 Is a Substrate of FTase

**FIGURE 3:**
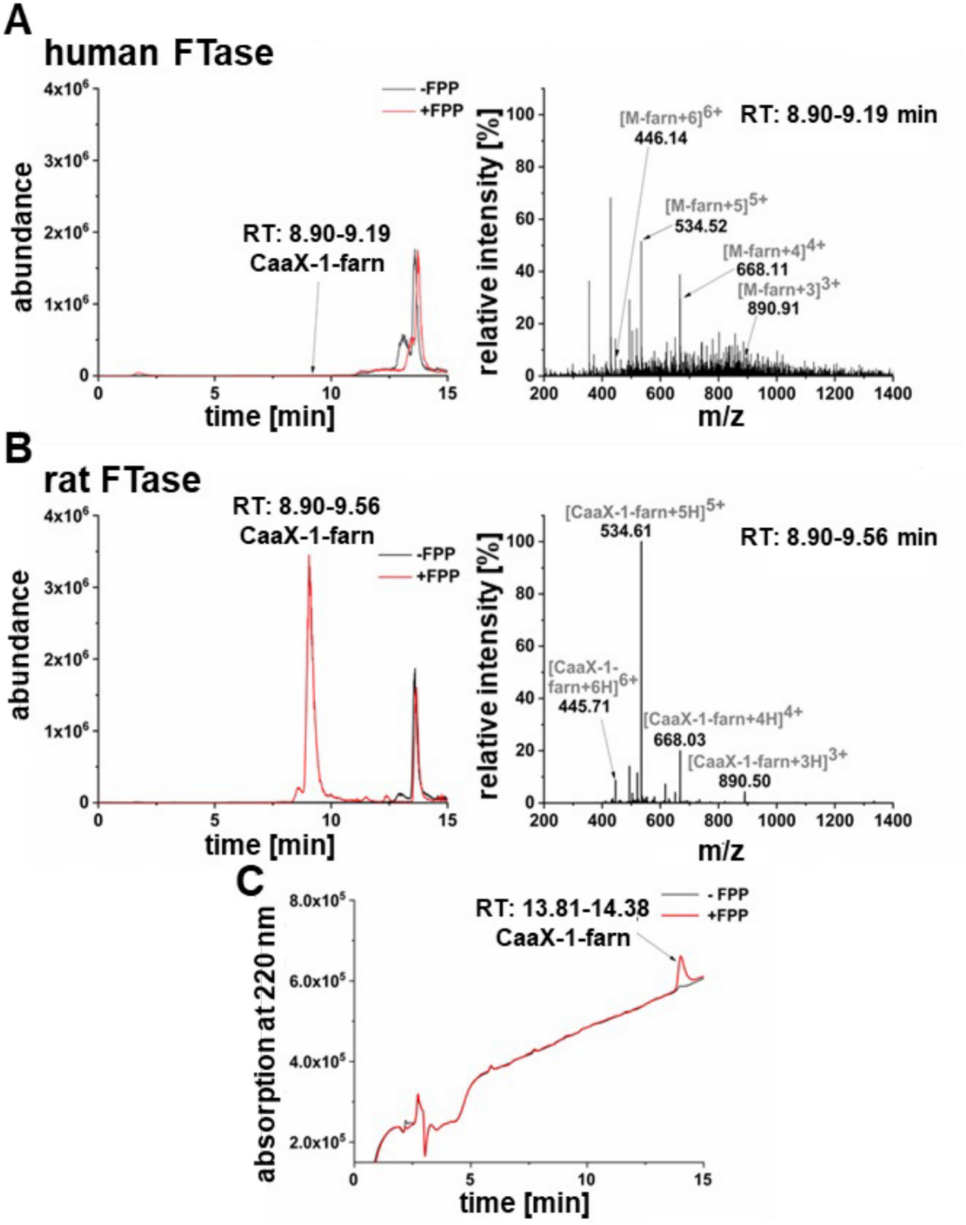
LC‐ESI‐MS analysis of the farnesylation of CaaX‐1 using *human* (A) and *rat* (B) FTase. (A) Ion current of the reaction with and without FPP and the respective mass spectrum of the reaction with FPP at 8.90‐ to 9.19‐min retention time. (B) Ion current of the reaction with and without FPP and mass spectrum of the reaction with FPP at 8.80‐ to 9.56‐min retention time. A linear gradient of 10%/90%/0.1% ACN/H_2_O/FA in 15 min was used in (A) and (B). (C) UV chromatogram of the reaction by *r*FTase with and without FPP using a gradient of 10%/90%/0.1% ACN/H_2_O/TFA (v/v/v) in 15 min. MW (CaaX‐1): 2463.08 Da; MW (CaaX‐1‐farn): 2667.27.

Next, we addressed the question whether CaaX‐1 peptides would be farnesylated by FTase ex cellulo. For this, CaaX‐1 was incubated with *h*FTase and the natural substrate FPP at 37°C for 18 h. Subsequently, the reaction mixture was desalted by C18 ZipTips and analyzed via LC‐ESI‐MS. We observed in the mass spectra that CaaX‐1 was clearly farnesylated. However, the results revealed a rather low efficiency of the farnesylation reaction (Figure 3A). Possibly, this was due to partial degradation of *h*FTase or other factors that reduced the activity of the enzyme. Consequently, we used commercially available *r*FTase and indeed detected a significantly more efficient farnesylation of CaaX‐1. The UV chromatogram showed a clear peak in the sample supplemented with FPP, and the mass spectrum demonstrated signals corresponding to the quasimolecular ions of farnesylated CaaX‐1 (Figure 3B,C). Although *h*FTase was less active, both experiments let conclude a successful reaction between the peptide CaaX‐1 and the respective prenyltransferases.

To get a deeper insight into the kinetics of this reaction, we then aimed to establish a fluorescence‐based interaction assay adapted to the work of Pompliano et al. [49]. Therefore, we first synthesized peptides bearing a dansyl group closely to the CaaX motif, for example, at the lysine side chain adjacent to the cysteine of the CaaX box. After interaction with FTase and farnesylation, the hydrophobic interaction between the dansyl and the farnesyl group would result in a shift of the fluorescence maximum detectable at 505 nm (Figure S6A) [49]. Furthermore, two control peptides were prepared, a positive control, namely, dan‐GCVLS, and a negative control, namely, dan‐GSVLS. Both of these short peptides have been already used for this assay [49]. Actually, when dan‐GCVLS was incubated with *r*FTase, a strong increase in fluorescence during the first 100 min of measurement was observed indicating a high conversion rate of the peptide. In contrast, not only the negative control peptide dan‐GSVLS but surprisingly also dan‐CaaX‐1 did not yield any increase in fluorescence when performing the same experiment (Figures S6B and S7). Maybe this finding was associated with the length of CaaX‐1 because in previous studies, only short peptides containing not more than five amino acids were utilized. The additional presence of 15 amino acids near to the dansyl modification potentially interfered with the hydrophobic interaction between the dansyl and farnesyl group. Moreover, the helical wheel projection of dan‐CaaX‐1 assumed that in the case of a successful farnesylation, the lipid chain would be likely too far away from the dansyl group for having any impact on the fluorescence (Figure S8). In addition, the dansyl group was already placed near hydrophobic amino acids (in CaaX‐1), which probably led to direct hydrophobic interactions. This may be why we observed relatively high levels of fluorescence for CaaX‐1 already at the beginning of this assay. Concluding, we were not able to transfer the system to CaaX‐1, which is why we omitted to establish this assay further.

**FIGURE 4:**
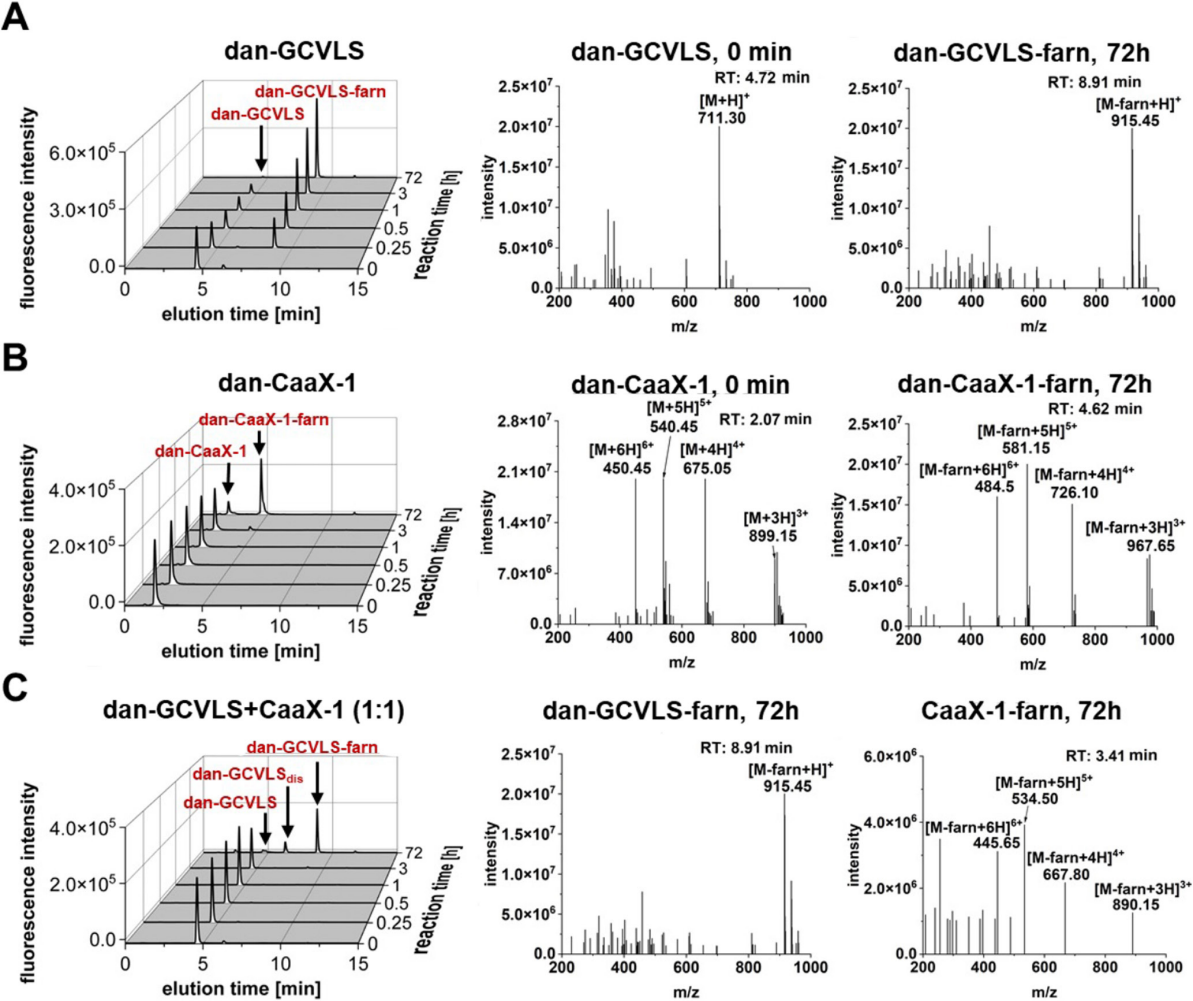
Time‐dependent LC‐ESI‐MS analysis of the farnesylation of (A) dan‐GCVLS, (B) dan‐CaaX‐1, and (C) a combination of dan‐GCVLS and CaaX‐1 using *r*FTase. For analysis, a linear gradient of 10–60 ACN in H_2_O with 0.1% FA in 15 min was used for liquid chromatography and a fluorescence detector (*λ*_ex_ = 340 nm, *λ*_em_ = 505 nm) with a subsequent ESI‐mass spectrometer was used for detection. Additional mass spectra of dan‐GCVLS (0 min), dan‐GCVLS_dis_ (72 h), and CaaX‐1 (0 min) corresponding to (C) are shown in Figure S8.

**FIGURE 5:**
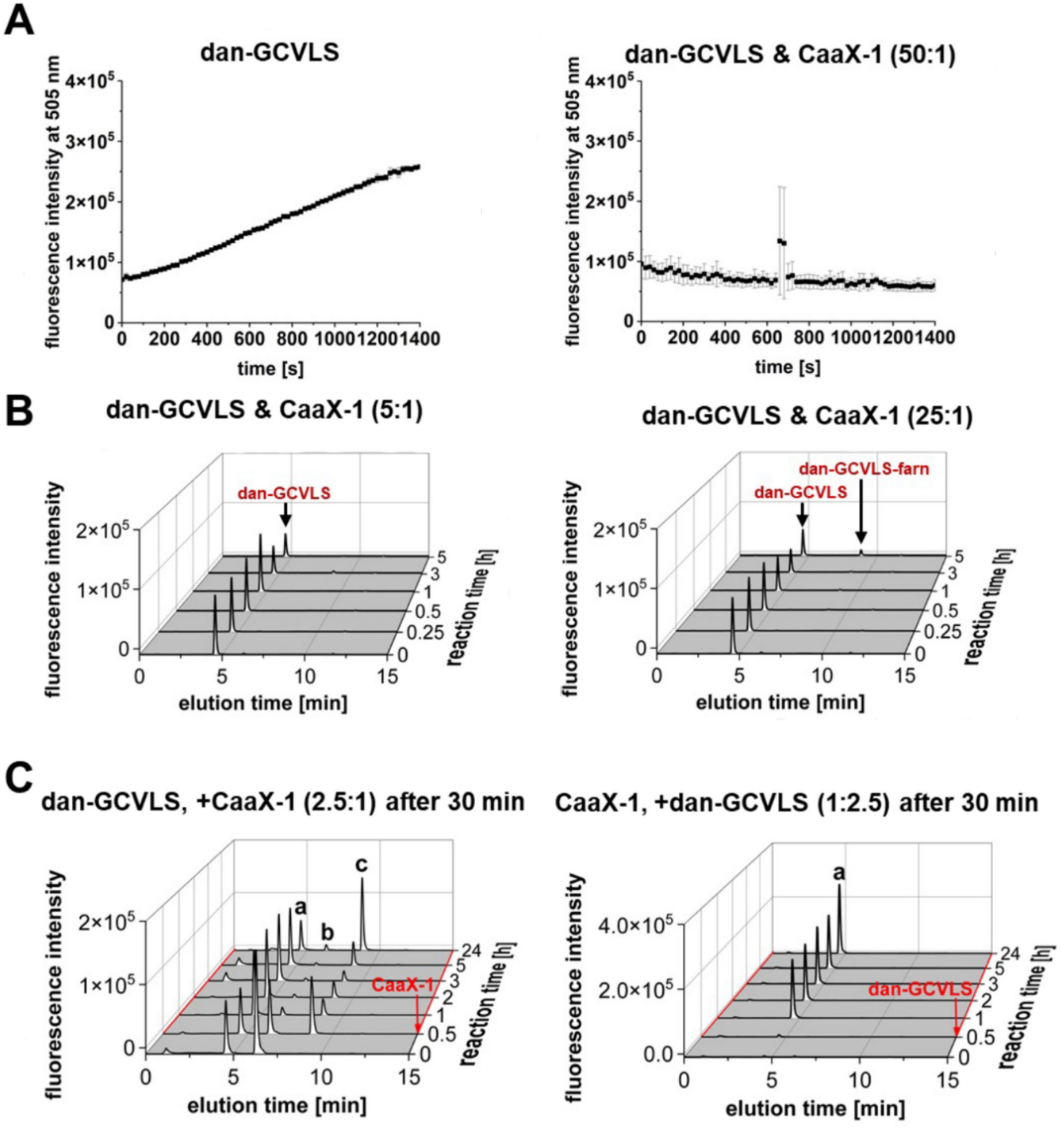
(A) Dansyl‐based farnesylation competition assay by coincubating dan‐GCVLS and CaaX‐1 (50:1) with *r*FTase and FPP and measuring the fluorescence (*λ*_ex_ = 340 nm, *λ*_em_ = 505 nm). Measurement was performed in three technical replicates. (B) Time‐dependent LC‐ESI‐MS analysis of the farnesylation competition assay of different ratios of dan‐GCVLS and CaaX‐1. The respective mass spectra are shown in Figure S9. (C) Time‐dependent LC‐ESI‐MS analysis of the farnesylation competition assay with preincubation of CaaX‐1 or dan‐GCVLS. Addition of the respective peptide after 30 min is indicated by a red arrow. (a) dan‐GCVLS, (b) dan‐GCVLS_dis_, and (c) dan‐GCVLS‐farn. For (B) and (C), a linear gradient of 10%–60% ACN in H_2_O + 0.1% FA in 15 min was used (*λ*_ex_ = 340 nm, *λ*_em_ = 505 nm).

We next compared the conversion of dan‐CaaX‐1 and dan‐GCVLS with *r*FTase using an LC‐ESI‐MS approach. Notably, dan‐GCVLS was quickly farnesylated; after 15 min, already half of it was prenylated, and after 3 h, only a small amount of dan‐GCVLS was left (Figure 4A). In contrast, dan‐CaaX‐1 was farnesylated significantly slower by *r*FTase because farnesylated dan‐CaaX‐1 was first detected after 3 h, and after 72 h, unfarnesylated peptide was still visible (Figure 4B). However, in our previous work, we performed pull‐down assays and observed an interaction of CaaX‐1 with FTase after only 2 h of incubation [48]. This suggested that CaaX‐1 probably already interacts with FTase considerably before any farnesylation event can be detected. If so, CaaX‐1 potentially influences farnesylation of other proteins by competing with them for binding to FTase. Therefore, we investigated whether CaaX‐1 is able to inhibit farnesylation of the positive control peptide. Indeed, coincubation of dan‐GCVLS and CaaX‐1 (1:1) with *r*FTase and FPP obviously inhibited farnesylation of dan‐GCVLS, as dan‐GCVLS‐farn was not detected after 3 h (Figure 5C). Only after 72 h, farnesylation of dan‐GCVLS was measured. Moreover, farnesylation of dan‐GCVLS was slowed down so much that even disulfides of dan‐GCVLS were formed over time after consumption of the added DTT, probably because of a competition with the farnesylation reaction (Figures 4C and S9). However, also farnesylated CaaX‐1 was observed after 72 h. Overall, we concluded that CaaX‐1 binds strongly to the active site of FTase, and this interaction might be able to inhibit farnesylation of dan‐GCVLS for a certain time period.

### 3.4 CaaX‐1 Functions as a Competitor

Based on our findings thus far, we aimed to learn more about the efficiency of the observed competition reaction. Thus, we coincubated different ratios of dan‐GCVLS and CaaX‐1 with *r*FTase and FPP for 1400 s at 37°C and monitored the change in fluorescence of dan‐GCVLS (Figures 5A and S10A). As expected, CaaX‐1 completely inhibited farnesylation of dan‐GCVLS even when dan‐GCVLS was present in 50‐fold excess (Figure 5A). We confirmed this by using LC‐ESI‐MS, too, because this method allowed us to analyze the abundant species during the farnesylation process more precisely. In fact, performing the assay in a 5:1 ratio of dan‐GCVLS and CaaX‐1 for up to 5 h completely prevented the conversion to dan‐GCVLS‐farn (Figure 5B). Only at a 25‐fold lower concentration of CaaX‐1, a very small amount of farnesylated dan‐GCVLS was detectable after 5 h. This again supported the assumption that CaaX‐1 interacts with FTase and that this interaction hinders the interaction of the positive control peptide with FTase and consequently, its farnesylation. Moreover, CaaX‐1 was farnesylated by FTase after a while (Figure 4B).

In a next experiment, we investigated whether CaaX‐1 is able to directly compete and thus to replace dan‐GCVLS from the active site of FTase. For this, dan‐GCVLS was pretreated with *r*FTase and FPP at 37°C and after 30 min, CaaX‐1 (2.5:1) was added. Before the addition of CaaX‐1, the active site of FTase should be freely accessible, and indeed, farnesylated dan‐GCVLS was detected after 30 min (Figure 5C). However, after addition of CaaX‐1, no further increase in farnesylation of dan‐GCVLS was visible, and it seemed that the reaction has slowed down for up to 5 h suggesting higher affinity of CaaX‐1 to FTase. This would reflect that FTase has a 50‐fold higher affinity for KRas4B, from which CaaX‐1 is derived, than for HRas having the CVLS CaaX motif [44]. Notably, this selectivity of FTase for the CaaX motif was not affected by the further attached amino acids that were derived from the CPP sC18*. Only after 24 h, further farnesylation of dan‐GCVLS was measured. Given these results, we wondered whether farnesylation of dan‐GCVLS would occur if we would preincubate *r*FTase with CaaX‐1 and FPP so that the active site of FTase would be already saturated by CaaX‐1. Indeed, no farnesylation of dan‐GCVLS was detected for up to 24 h, supporting the hypothesis of a higher affinity of CaaX‐1 to FTase.

### 3.5 Relevance of the CaaX Motif for FTase Interaction and Biological Activity

**FIGURE 6:**
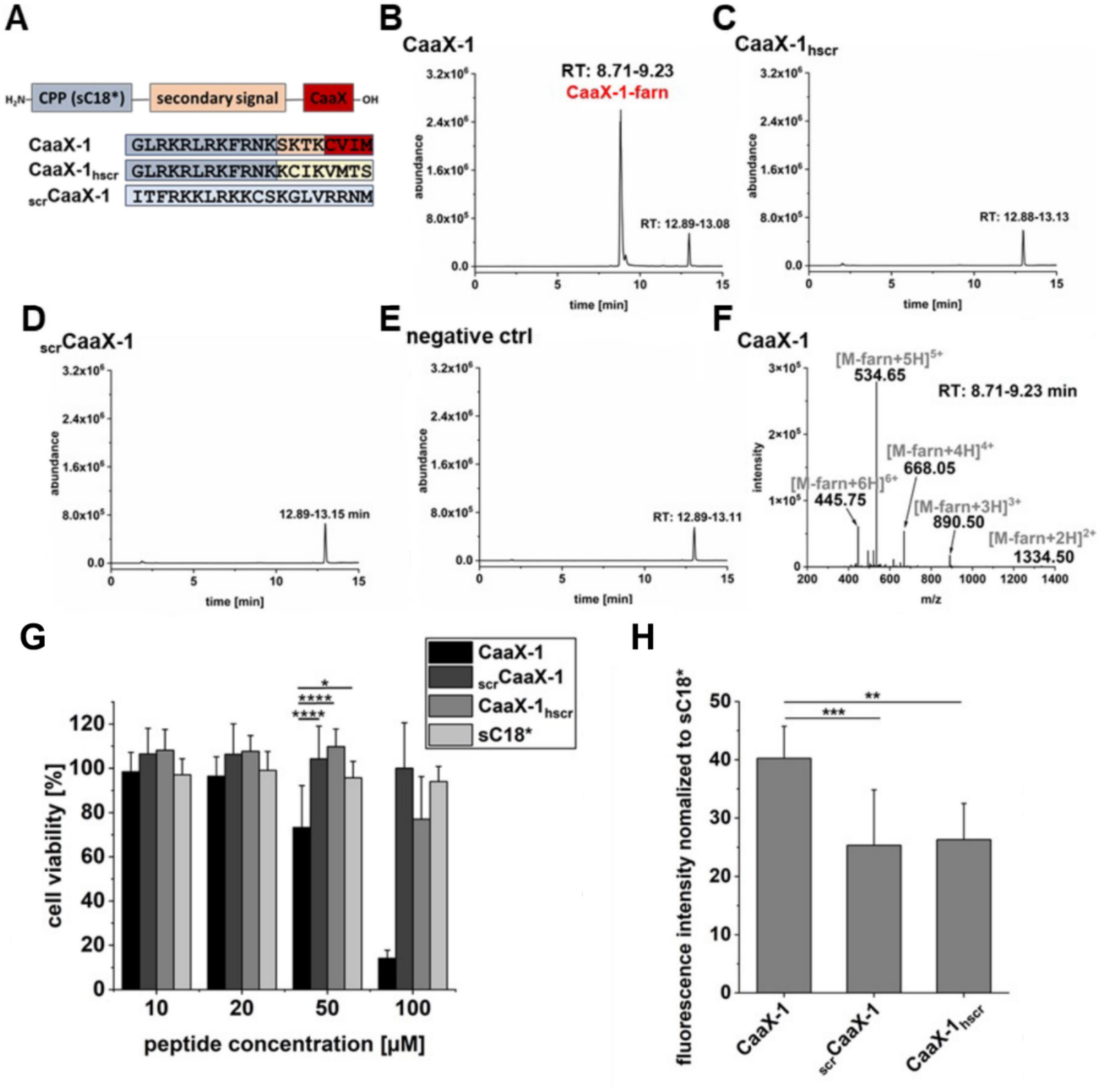
(A) Design of different scrambled controls of CaaX‐1. (B–E) Ion currents of the LC–MS analysis (linear gradient of 10%–60% ACN in H_2_O + 0.1% FA in 15 min) of the reactions of CaaX‐1 or its scrambled controls with *r*FTase and FPP. (F) Mass spectrum of the peak at 8.71–9.23 min of the ion current in (B). (G) Cytotoxicity of the scrambled control peptides at different concentrations to HeLa cells. Viability of untreated cells were set to 100%. Measurement was performed in triplicate (*n* = 3). (H) Cellular uptake of the scrambled control peptides (10 μM) in HeLa cells after 30 min. Measurement was performed in triplicate (*n* = 4). Significances were calculated by (G) two‐way ANOVA or (H) one‐way ANOVA (*p < 0.05, **p < 0.01, ***p < 0.001), and standard deviations are represented by error bars.

Finally, we examined the relevance of an intact CaaX motif ex cellulo and in cellular studies. For this, we synthesized control peptides including CaaX‐1_hscr_ in which the secondary signal and the CaaX motif were scrambled and _scr_CaaX‐1 having a complete scrambled sequence of CaaX‐1 (Figure 6A). First, we tested if farnesylation would occur when incubating each peptide for 18 h at 37°C with *r*FTase. Notably, analysis by LC‐ESI‐MS demonstrated that only CaaX‐1 was farnesylated by *r*FTase highlighting the importance of an intact CaaX motif for this reaction (Figure 6B–F). Moreover, AlphaFold3 predictions of the interaction of FTase with the scrambled control peptides indicated an altered localization of the peptides within the active site of FTase compared to CaaX‐1, as the cysteine residue of the scrambled peptides is too far away from the zinc ion within the active site, supporting the results of a lack of catalytic conversion of these peptides (Figure S12).

Then, we compared the cytotoxic effects of the scrambled controls and CaaX‐1 when incubated with HeLa cells. Interestingly, both scrambled versions exhibited nearly no cytotoxic effects, only CaaX‐1_hscr_ revealed slight toxicity when applied at 100 μM (Figure 6G). Also of note is that both scrambled peptides were efficiently internalized, although to slightly less extent compared to CaaX‐1. However, in our opinion, this clearly indicated that the influence on cell viability of CaaX‐1 was dependent on an intact CaaX motif and that this is also critical for efficient internalization of CaaX‐1 (Figure 6H).

## 4 Conclusion

Recently, we developed cell‐permeable CaaX peptides containing a CPP and a CaaX motif [48]. Interestingly, these peptides are characterized by a high cellular uptake, and previous studies let conclude that this intracellular accumulation is not mediated by thiol‐related uptake mechanisms [64]. Based on these findings, we proposed that cellular activity of the CaaX peptides was rather the result of CaaX peptides interfering with the farnesylation machinery and thus farnesylation of proteins such as Ras. Within this work, we aimed to gain more insights into the underlying mechanisms focusing on the interactions between FTase and CaaX‐1 peptides. We demonstrated that CaaX‐1 efficiently interacts with and becomes farnesylated by FTase ex cellulo and that an intact CaaX motif is required for receiving such a modification. Although ex cellulo farnesylation of a positive control peptide (GCVLS) was faster, CaaX‐1 was able to inhibit farnesylation of this peptide already at 25‐ to 50‐fold lower concentrations. This suggests that the conversion of CaaX‐1 to its farnesylated derivative is not as rapid. However, the affinity between CaaX‐1 and FTase appears to be relatively high, as even low concentrations of CaaX‐1 are sufficient to prevent farnesylation of the positive control peptide, which agrees to previous findings showing an approximately 50‐fold higher affinity of FTase for KRas4B than for HRas. This makes it a promising candidate as a competitor of proteins for farnesylation, and it may serve as a template for further inhibitors. However, one major drawback of FTIs is cross‐prenylation by GGTase I. As AlphaFold3 predictions indicated also interactions of CaaX‐1 with GGTase I (Figure S11), we will also investigate whether this peptide interacts with GGTase I and might interfere also with geranylgeranylation of positive control peptides by GGTase I. Furthermore, we demonstrated the dependence of the CaaX motif for efficient internalization in HeLa cells. This suggests that CaaX peptides are also prenylated by endogenous prenyltransferases and that this lipidation leads to increased cellular uptake, probably by removing CaaX‐1 from the internalization equilibrium. Taken together, cell‐permeable CaaX peptides are interesting tools to study and manipulate the prenylation process potentially of the whole prenylome.

## Conflicts of Interest

The authors declare no conflicts of interest.

## Supporting information


**Figure S1.** LC–MS analysis of (A) dan‐CaaX‐1, (B) dan‐control_pos_, and (C) dan‐control_neg_ using a gradient of (A) 10%–60% ACN in water and 0.1% TFA or (B,C) 20%–70% ACN in water and 0.1% TFA in 15 min.
**Figure S2.** LC–MS analysis of (A) CaaX‐1 and (B) CF‐CaaX‐1 using a gradient of 10%–60% ACN in water and 0.1% TFA in 15 min.
**Figure S3.** LC–MS analysis of peptides _scr_CaaX‐1 (A) and CaaX‐1_hscr_ (B) using a gradient of 10%–60% ACN in water and 0.1% FA in 15 min.
**Figure S4.** LC–MS analysis of the CF‐labeled scrambled controls of CaaX‐1 using a gradient of 10%–60% ACN in water and 0.1% FA in 15 min.
**Figure S5.** LC–MS analysis of (A) sC18* and (B) CF‐sC18* using a gradient of 10%–60% ACN in water and 0.1% FA in 15 min.
**Figure S6.** Fluorescence‐based farnesylation assay. (A) Design of the dansyl‐based farnesylation assay. (B) *r*FTase was incubated with 50‐μM dan‐GCVLS, dan‐GSVLS, or dan‐CaaX‐1 and 10‐μM FPP and fluorescence intensity at 505 nm was measured after excitation at 340 nm for 200 min. Measurement was performed in three technical replicates.
**Figure S7.** Fluorescence‐based farnesylation assay. *Human* FTase was incubated with 50‐μM dan‐GCVLS, dan‐GSVLS, or dan‐CaaX‐1 and 10‐μM FPP and fluorescence intensity at 505 nm was measured after excitation at 340 nm for 200 min. Measurement was performed in three technical replicates.
**Figure S8.** Helical wheel projection of dan‐CaaX‐1 [1] (accessed on August 8, 2023).
**Figure S9.** Mass spectra of the LC–MS measurement shown in Figure 4C at retention times 1.37, 4.72, and 6.46 min.
**Figure S10.** (A) Control measurements of the dansyl‐based farnesylation competition assay shown in Figure 5A. Measurement was performed in three technical replicates and error bars represent standard deviation. (B) Time‐dependent LC–MS analysis of the competition for farnesylation of different ratios of dan‐GCVLS and CaaX‐1 compared to the measurements shown in Figure 5B using rat FTase and FPP. (C) Mass spectra of the LC–MS analysis of the experiment shown in Figure 5B showing dan‐GCVLS (left) and dan‐GCVLS‐farn (right).
**Figure S11.** (A) AlphaFold3 prediction of the interactions between CaaX‐1 (yellow) and the zinc ion (grey) complexed by the active site of *h*GGTase I (cyan). AlphaFold3 prediction was processed and modified with PyMOL using PDB 1N4P. The zinc ion was inserted with Coot. (B) Overview of the predicted localization of CaaX‐1 within GGTase I. CaaX‐1 localizes with its CaaX motif within the active site of FTase in the interface between the α‐subunit and the β‐subunit. Predicted interactions of CaaX‐1 with the (C) α‐subunit or (D) the β‐subunit of GGTase I. Dark blue highlights highly reliable interactions, light blue reliable interactions, yellow more less reliable interactions, and red very less reliable interactions. Images were made with ChimeraX [2–4].
**Figure S12.** AlphaFold3 prediction of potential interactions between (A) CaaX‐1_hscr_ or (B) _scr_CaaX‐1 (peptides: yellow) and the zinc ion (grey) complexed by the active site of *h*GGTase I (cyan) suggesting no catalytic processing of the peptides due to far distance between catalytic site and the peptide's cysteine residue. AlphaFold3 prediction was processed and modified with PyMOL. The zinc ion was inserted with Coot.
**Table S1.** Peptides synthesized and used within this work. CF: 5(6)‐carboxyfluorescein; dan: dansyl.

## Data Availability

The data that support the findings of this study are available from the corresponding author upon reasonable request.
