# Supplementary material for: Examining Farnesyltransferase Interaction With Cell‐Permeable CaaX Peptides and the Role of the CaaX Motif in Biological Activity
Source: J Pept Sci. 2025 Mar 10;31(4):e70009. doi: 10.1002/psc.70009 (PMC11893521; doi:10.1002/psc.70009)
Supplement: Supplementary file 1 — Figure S1. LC–MS analysis of (A) dan‐CaaX‐1, (B) dan‐controlpos, and (C) dan‐controlneg using a gradient of (A) 10%–60% ACN in water and 0.1% TFA or (B,C) 20%–70% ACN in water and 0.1% TFA in 15 min. Figure S2. LC–MS analysis of (A) CaaX‐1 and (B) CF‐CaaX‐1 using a gradient of 10%–60% ACN in water and 0.1% TFA in 15 min. Figure S3. LC–MS analysis of peptides scrCaaX‐1 (A) and CaaX‐1hscr (B) using a gradient of 10%–60% ACN in water and 0.1% FA in 15 min. Figure S4. LC–MS analysis of the CF‐labeled scrambled controls of CaaX‐1 using a gradient of 10%–60% ACN in water and 0.1% FA in 15 min. Figure S5. LC–MS analysis of (A) sC18* and (B) CF‐sC18* using a gradient of 10%–60% ACN in water and 0.1% FA in 15 min. Figure S6. Fluorescence‐based farnesylation assay. (A) Design of the dansyl‐based farnesylation assay. (B) rFTase was incubated with 50‐μM dan‐GCVLS, dan‐GSVLS, or dan‐CaaX‐1 and 10‐μM FPP and fluorescence intensity at 505 nm was measured after excitation at 340 nm for 200 min. Measurement was performed in three technical replicates. Figure S7. Fluorescence‐based farnesylation assay. Human FTase was incubated with 50‐μM dan‐GCVLS, dan‐GSVLS, or dan‐CaaX‐1 and 10‐μM FPP and fluorescence intensity at 505 nm was measured after excitation at 340 nm for 200 min. Measurement was performed in three technical replicates. Figure S8. Helical wheel projection of dan‐CaaX‐1 [1] (accessed on August 8, 2023). Figure S9. Mass spectra of the LC–MS measurement shown in Figure 4C at retention times 1.37, 4.72, and 6.46 min. Figure S10. (A) Control measurements of the dansyl‐based farnesylation competition assay shown in Figure 5A. Measurement was performed in three technical replicates and error bars represent standard deviation. (B) Time‐dependent LC–MS analysis of the competition for farnesylation of different ratios of dan‐GCVLS and CaaX‐1 compared to the measurements shown in Figure 5B using rat FTase and FPP. (C) Mass spectra of the LC–MS analysis of the experiment shown in Fig [file PSC-31-e70009-s001.docx]

**Supporting Information**

**Examining farnesyltransferase interaction with CaaX-peptides and the role of the CaaX motif in biological activity**

Merlin Klußmann, Jan Reuter, Christian Werner, Ines Neundorf

*Institute for Biochemistry, University of Cologne, Zuelpicher Str. 47a, 50674 Cologne, Germany*

**Corresponding author:** Prof. Dr. Ines Neundorf, phone: +49 (0) 221 470 8847, e-mail: ines.neundorf@uni-koeln.de


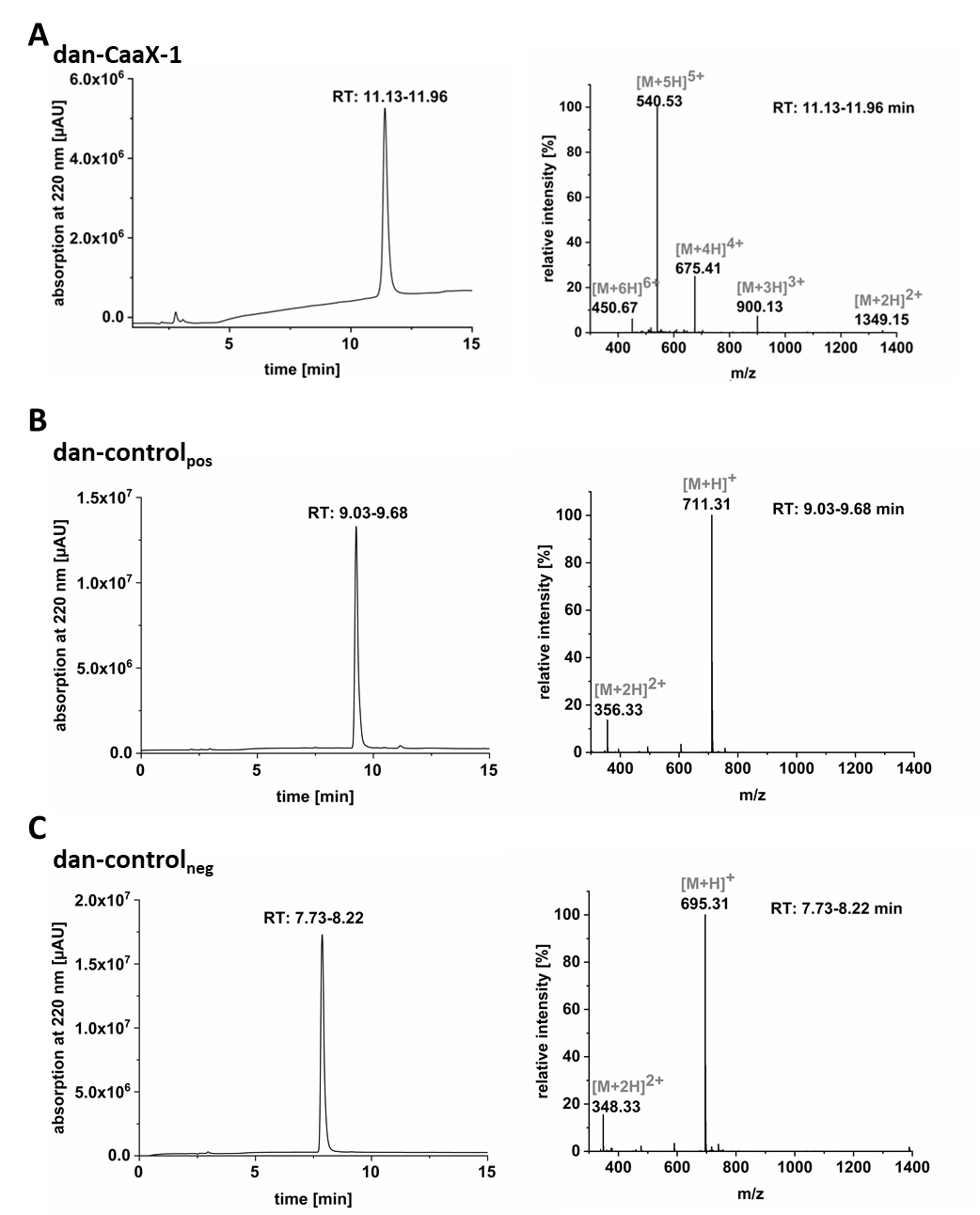


**Figure S1:** LC-MS analysis of (A) dan-CaaX-1, (B) dan-control_pos_, and (C) dan-control_neg_ using a gradient of (A) 10-60 % ACN in water and 0.1 % TFA or (B,C) 20-70 % ACN in water and 0.1 % TFA in 15 min.


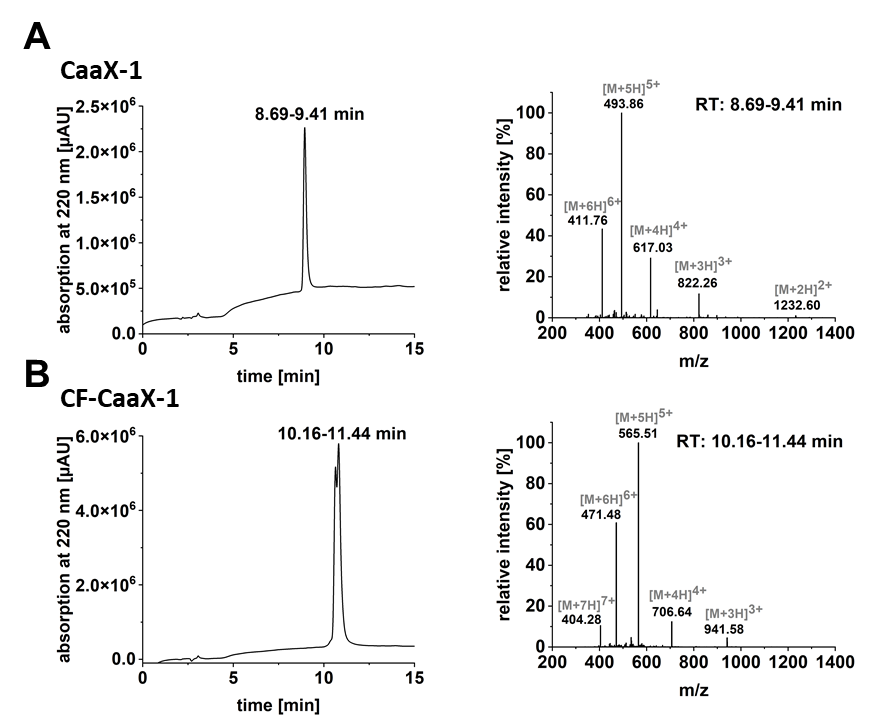


**Figure S2:** LC-MS analysis of (A) CaaX-1, and (B) CF-CaaX-1 using a gradient of 10-60 % ACN in water and 0.1 % TFA in 15 min.

**
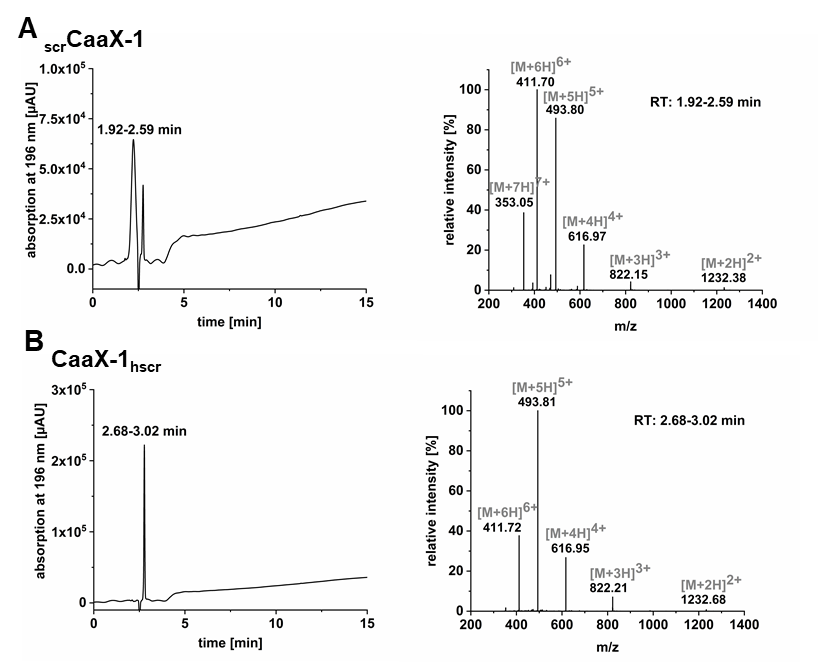
**

**Figure S3:** LC-MS analysis of peptides _scr_CaaX-1 (A) and CaaX-1_hscr_ (B) using a gradient of 10-60 % ACN in water and 0.1 % FA in 15 min.


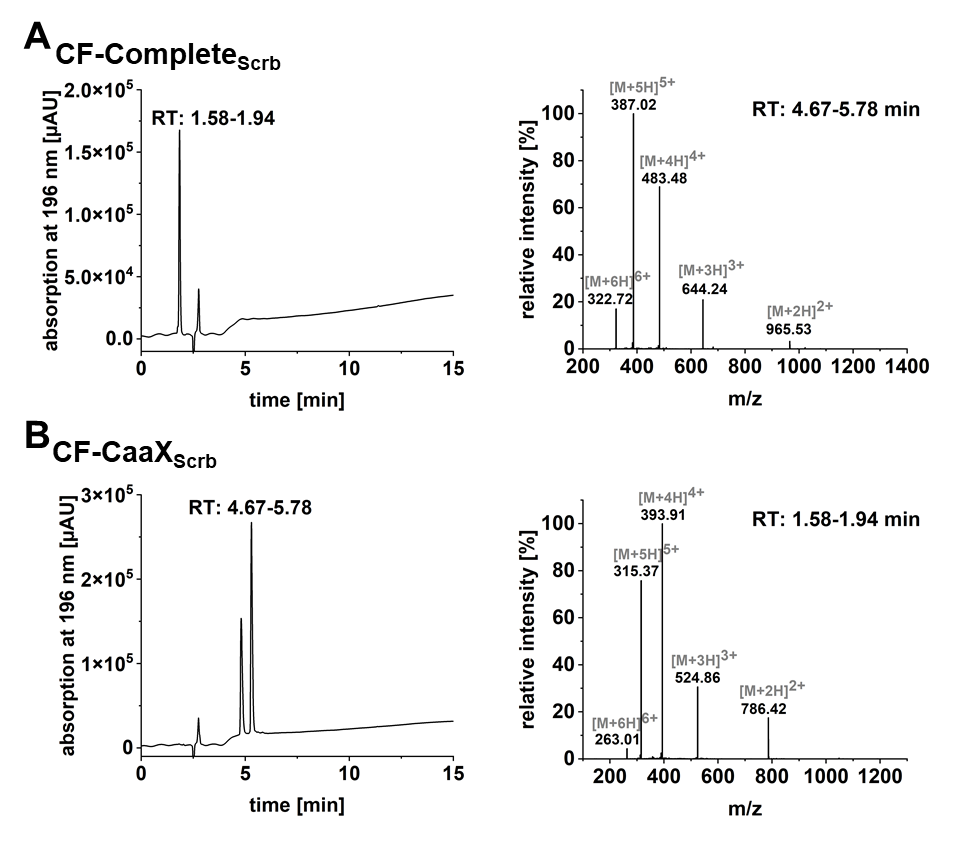


**Figure S4:** LC-MS analysis of the CF-labeled scrambled controls of CaaX-1 using a gradient of 10-60 % ACN in water and 0.1 % FA in 15 min.


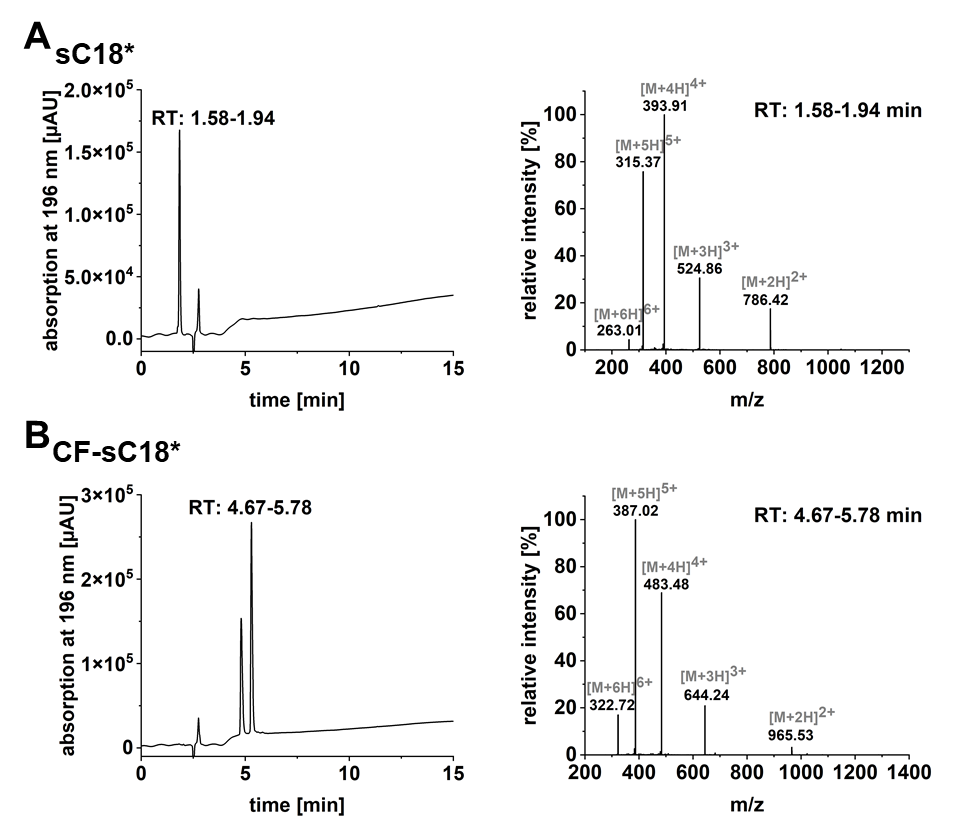


**Figure S5:** LC-MS analysis of (A) sC18* and (B) CF-sC18* using a gradient of 10-60 % ACN in water and 0.1 % FA in 15 min.


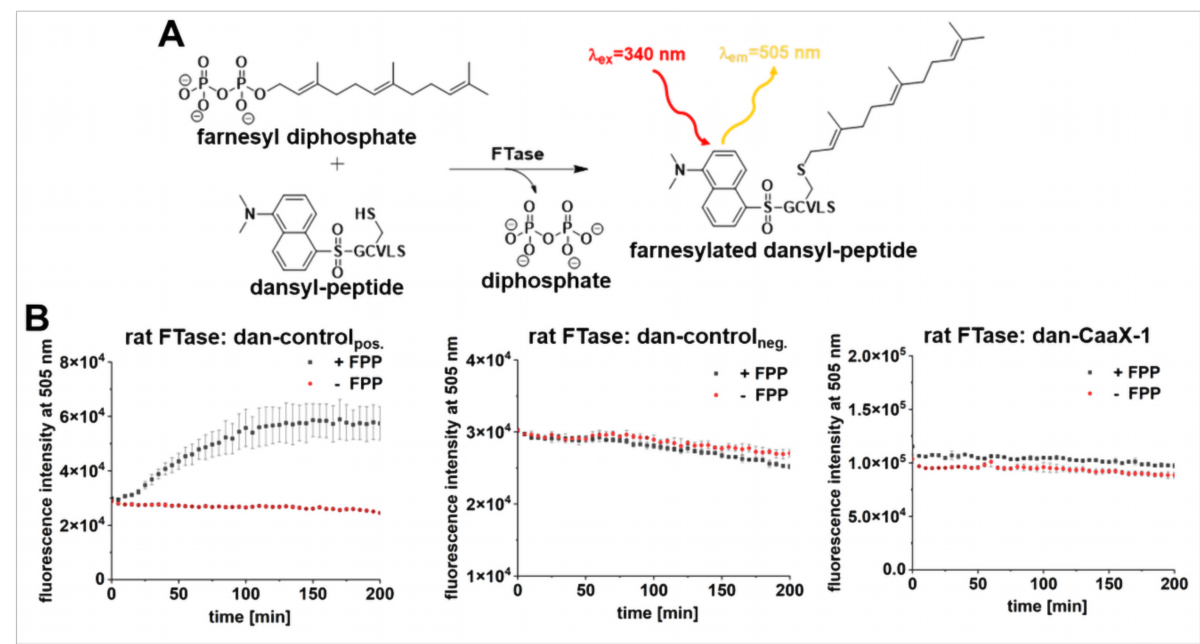


**Figure S6:** Fluorescence-based farnesylation assay. (A) Design of the dansyl-based farnesylation assay. (B) *r*FTase was incubated with 50 µM dan-GCVLS, dan-GSVLS, or dan-CaaX-1 and 10 µM FPP and fluorescence intensity at 505 nm was measured after excitation at 340 nm for 200 min. Measurement was performed in three technical replicates.


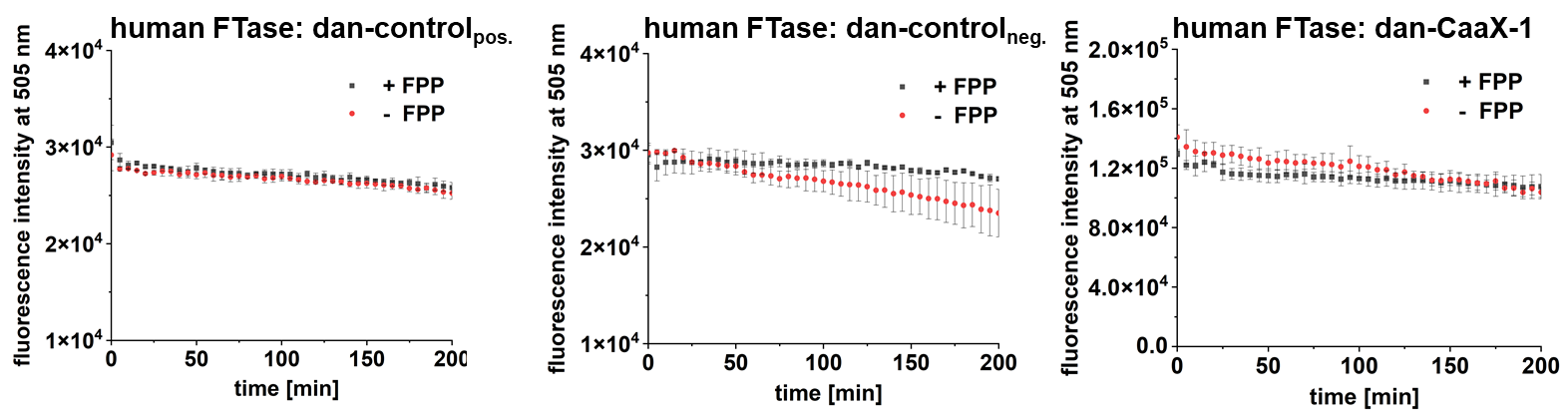


**Figure S7:** Fluorescence-based farnesylation assay. *Human* FTase was incubated with 50 µM dan-GCVLS, dan-GSVLS, or dan-CaaX-1 and 10 µM FPP and fluorescence intensity at 505 nm was measured after excitation at 340 nm for 200 min. Measurement was performed in three technical replicates.


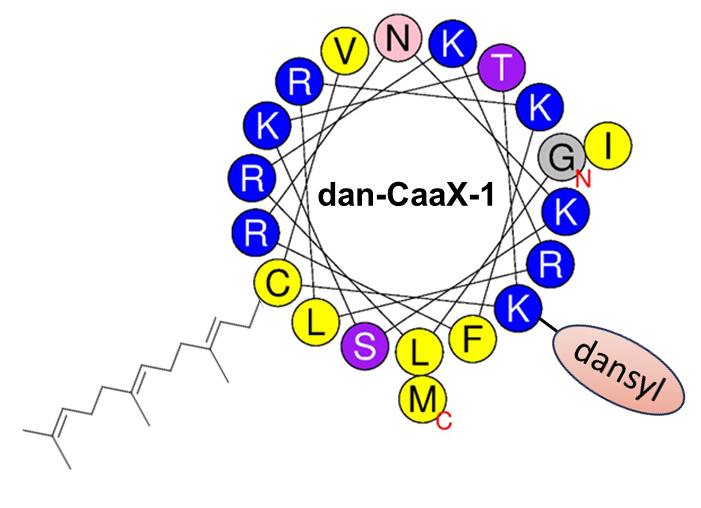


**Figure S8:** Helical wheel projection of dan-CaaX-1^1^ (accessed on Aug 8, 2023)).


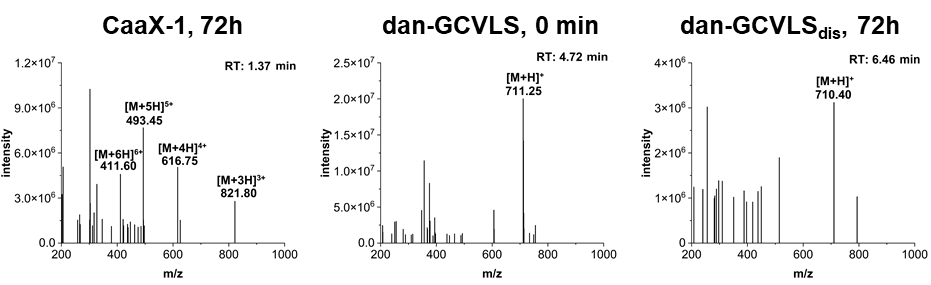


**Figure S9:** Mass spectra of the LC-MS measurement shown in Fig. 4C at retention times 1.37 min, 4.72 min, and 6.46 min.


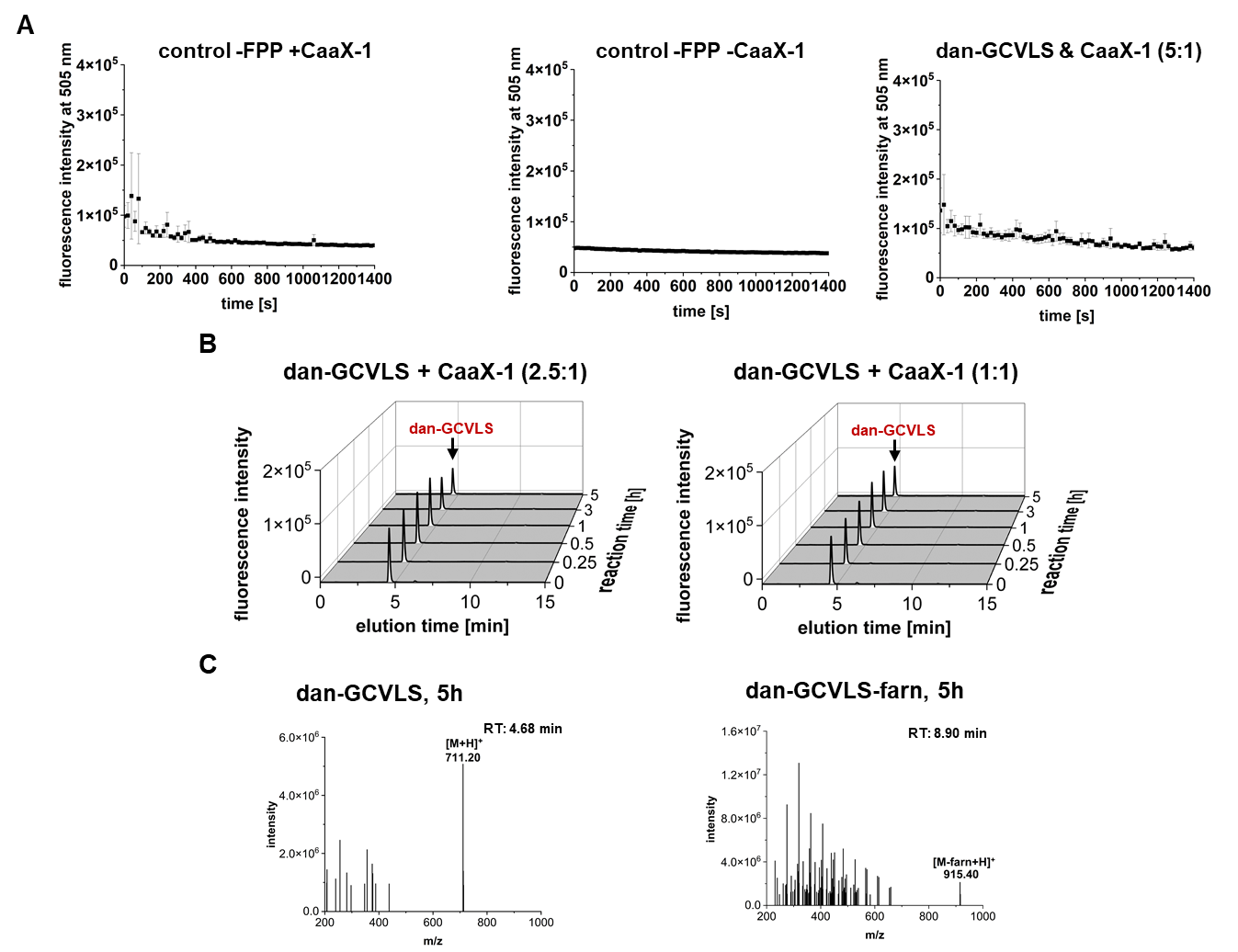


**Figure S10:** (A) Control measurements of the dansyl-based farnesylation competition assay shown in Figure 6A. Measurement was performed in three technical replicates and error bars represent standard deviation. (B) Time-dependent LC-MS analysis of the competition for farnesylation of different ratios of dan-GCVLS and CaaX-1 compared to the measurements shown in Figure 6B using rat FTase and FPP. (C) Mass spectra of the LC-MS analysis of the experiment shown in Figure 6B showing dan-GCVLS (left) and dan-GCVLS-farn (right).


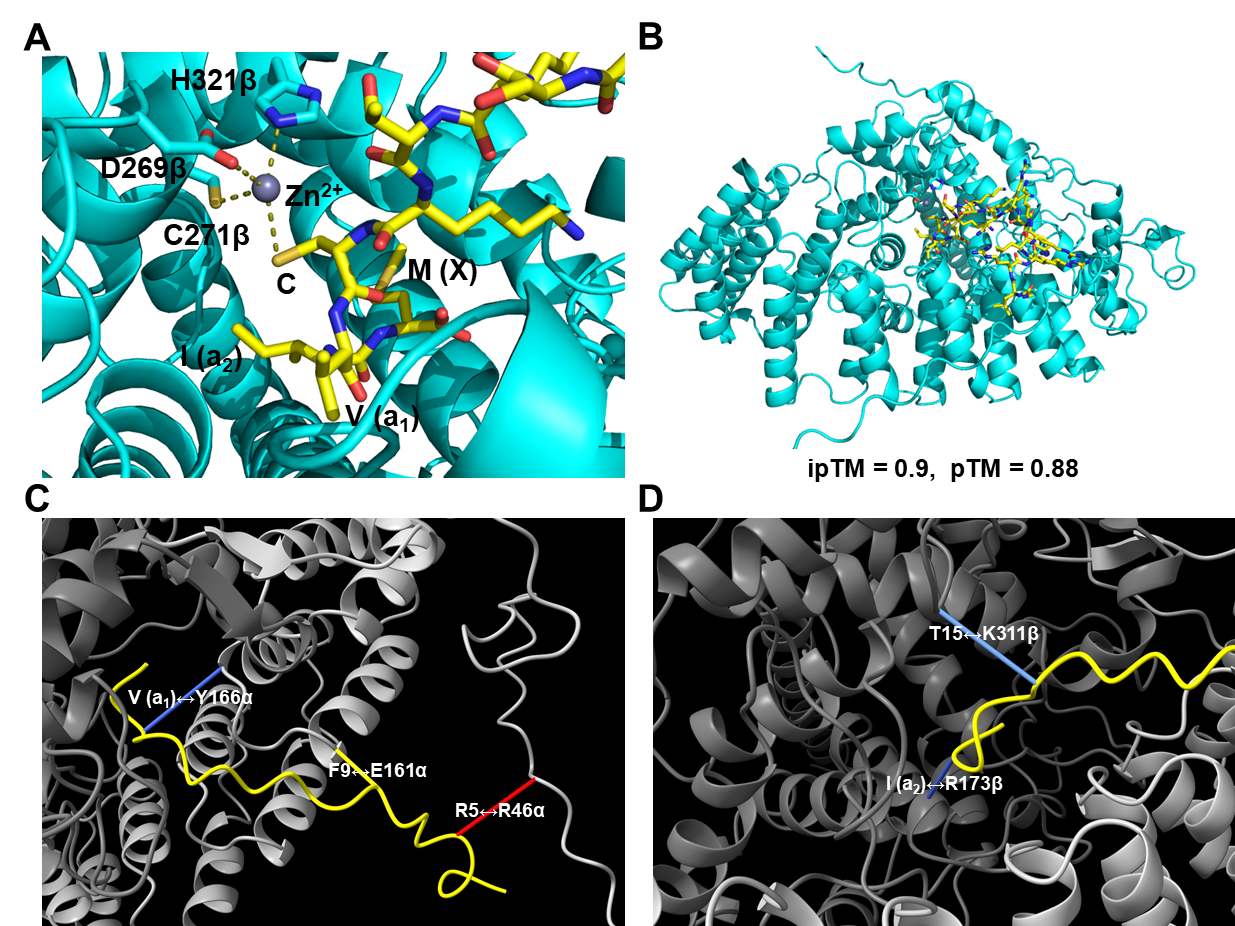


**Figure S11:** (A) AlphaFold3 prediction of the interactions between CaaX-1 (yellow) and the zinc ion (grey) complexed by the active site of *h*GGTase I (cyan). AlphaFold 3 prediction was processed and modified with PyMOL using PDB 1N4P. The zinc ion was inserted with Coot. (B) Overview of the predicted localization of CaaX-1 within GGTase I. CaaX-1 localizes with its CaaX motif within the active site of FTase in the interface between the α-subunit and the β-subunit. Predicted interactions of CaaX-1 with the (C) α-subunit or (D) the β-subunit of GGTase I. Dark blue highlights highly reliable interactions, light blue reliable interactions, yellow more less reliable interactions, and red very less reliable interactions. Images were made with ChimeraX.^2–4^


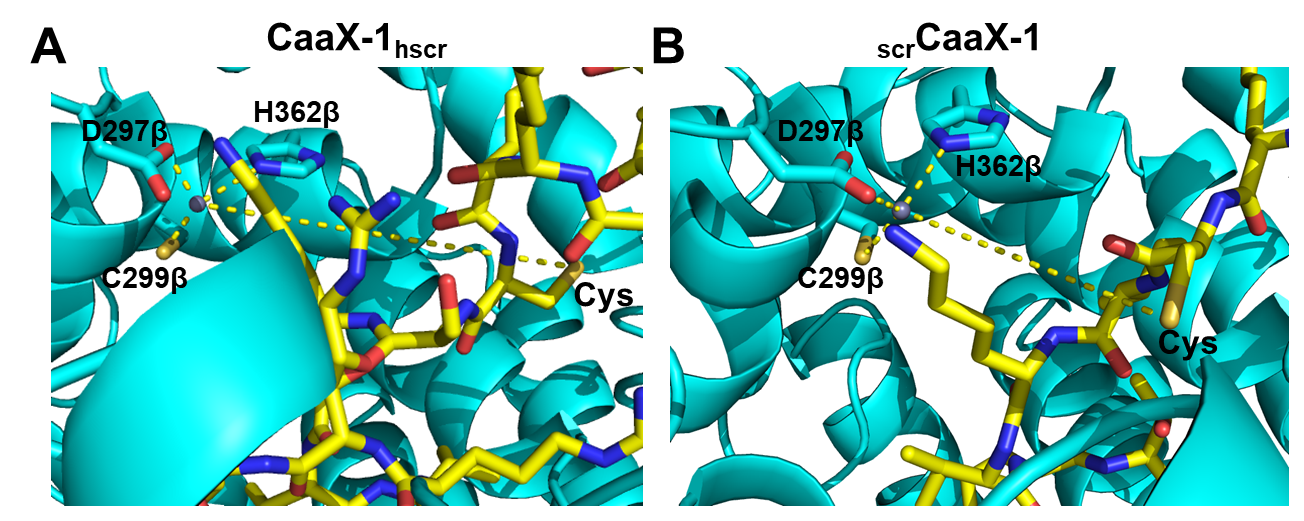


**Figure S12:** AlphaFold3 prediction of potential interactions between (A) CaaX-1_hscr_ or (B) _scr_CaaX-1 (peptides: yellow) and the zinc ion (grey) complexed by the active site of *h*GGTase I (cyan) suggesting no catalytic processing of the peptides due to far distance between catalytic site and the peptide’s cysteine residue. AlphaFold 3 prediction was processed and modified with PyMOL. The zinc ion was inserted with Coot.

**Table S1:** Peptides synthesized and used within this work. CF: 5(6)-carboxyfluorescein; dan: dansyl.


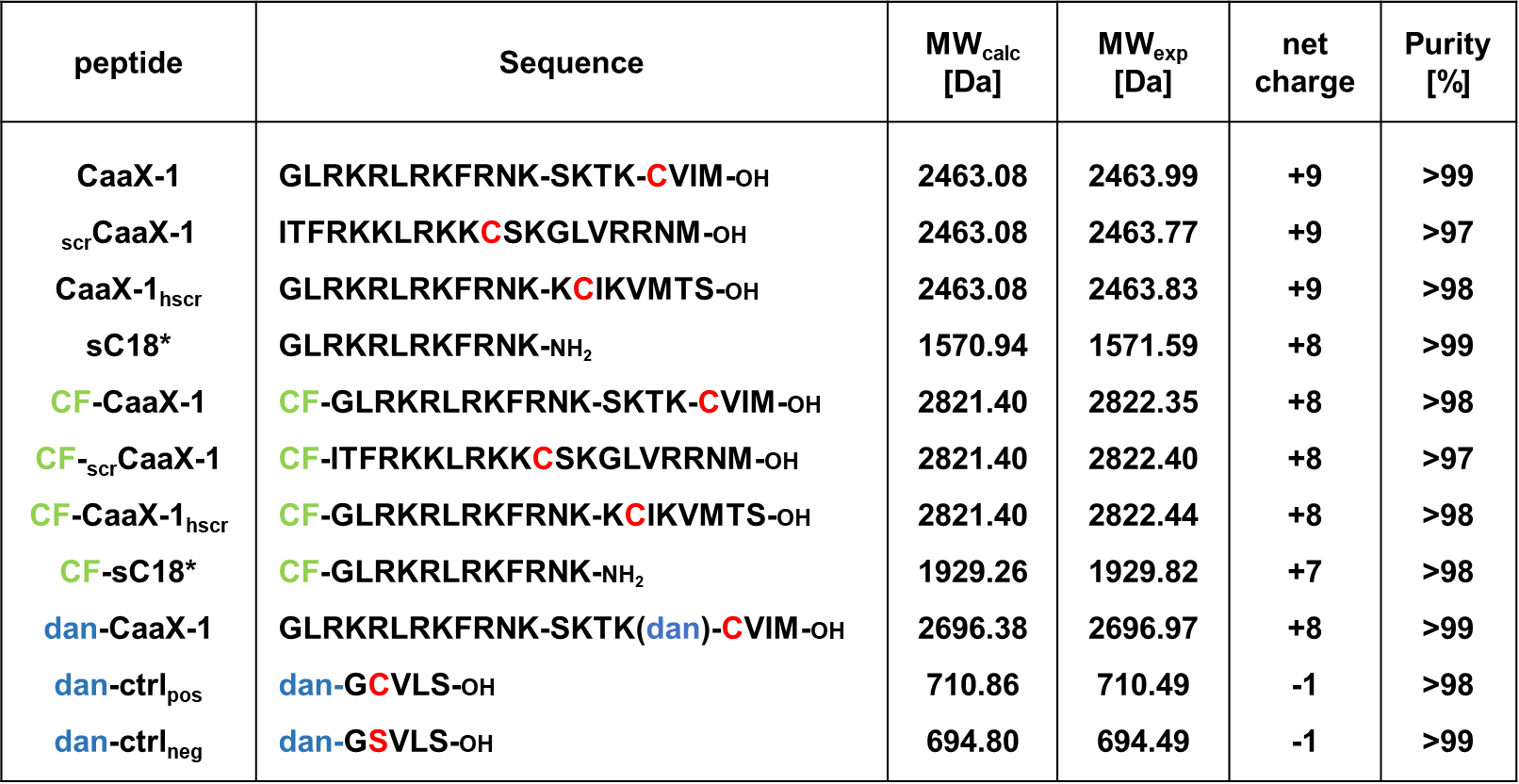


1. HeliQuest ComputParam form version3 https://heliquest.ipmc.cnrs.fr/cgi-bin/ComputParams.py (accessed Aug 8, 2023).

2. Meng EC, Goddard TD, Pettersen EF, Couch GS, Pearson ZJ, Morris JH, Ferrin TE. UCSF ChimeraX: Tools for Structure Building and Analysis. *Protein Sci* 2023;32(11):e4792, DOI: 10.1002/PRO.4792.

3. Goddard TD, Huang CC, Meng EC, Pettersen EF, Couch GS, Morris JH, Ferrin TE. UCSF ChimeraX: Meeting Modern Challenges in Visualization and Analysis. *Protein Sci* 2018;27(1):14–25, DOI: 10.1002/PRO.3235.

4. Pettersen EF, Goddard TD, Huang CC, Meng EC, Couch GS, Croll TI, Morris JH, Ferrin TE. UCSF ChimeraX: Structure Visualization for Researchers, Educators, and Developers. *Protein Sci* 2021;30(1):70–82, DOI: 10.1002/PRO.3943.
